# A Unified Framework for Understanding Nucleophilicity and Protophilicity in the S_N_2/E2 Competition

**DOI:** 10.1002/chem.202003831

**Published:** 2020-10-22

**Authors:** Pascal Vermeeren, Thomas Hansen, Paul Jansen, Marcel Swart, Trevor A. Hamlin, F. Matthias Bickelhaupt

**Affiliations:** ^1^ Department of Theoretical Chemistry Amsterdam Institute of, Molecular and Life Sciences (AIMMS) Amsterdam Center for Multiscale, Modeling (ACMM) Vrije Universiteit Amsterdam De Boelelaan 1083 1081 HV Amsterdam The Netherlands; ^2^ Leiden Institute of Chemistry Leiden University Einsteinweg 55, 2333 CC Leiden The Netherlands; ^3^ Laboratory of Physical Chemistry ETH Zurich Vladimir-Prelog-Weg 2 8093 Zurich Switzerland; ^4^ ICREA Pg. Lluís Companys 23 08010 Barcelona Spain; ^5^ IQCC & Dept. Química Universitat de Girona Campus Montilivi (Ciències) 17003 Girona Spain; ^6^ Institute for Molecules and Materials Radboud University Heyendaalseweg 135 6525 AJ Nijmegen The Netherlands

**Keywords:** activation strain model, bond theory, density functional calculations, nucleophilicity, protophilicity

## Abstract

The concepts of nucleophilicity and protophilicity are fundamental and ubiquitous in chemistry. A case in point is bimolecular nucleophilic substitution (S_N_2) and base‐induced elimination (E2). A Lewis base acting as a strong nucleophile is needed for S_N_2 reactions, whereas a Lewis base acting as a strong protophile (i.e., base) is required for E2 reactions. A complicating factor is, however, the fact that a good nucleophile is often a strong protophile. Nevertheless, a sound, physical model that explains, in a transparent manner, when an electron‐rich Lewis base acts as a protophile or a nucleophile, which is not just phenomenological, is currently lacking in the literature. To address this fundamental question, the potential energy surfaces of the S_N_2 and E2 reactions of X^−^+C_2_H_5_Y model systems with X, Y = F, Cl, Br, I, and At, are explored by using relativistic density functional theory at ZORA‐OLYP/TZ2P. These explorations have yielded a consistent overview of reactivity trends over a wide range in reactivity and pathways. Activation strain analyses of these reactions reveal the factors that determine the shape of the potential energy surfaces and hence govern the propensity of the Lewis base to act as a nucleophile or protophile. The concepts of “characteristic distortivity” and “transition state acidity” of a reaction are introduced, which have the potential to enable chemists to better understand and design reactions for synthesis.

## Graphical abstract

**It's about time**! A transparent and physically sound model is proposed to recognize when a Lewis base will react as a nucleophile (S_N_2) or protophile (E2) and the novel concepts of “characteristic distortivity” and “transition state acidity” are introduced.

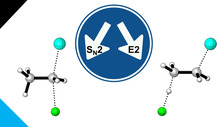

## Introduction

**Scheme 1:**
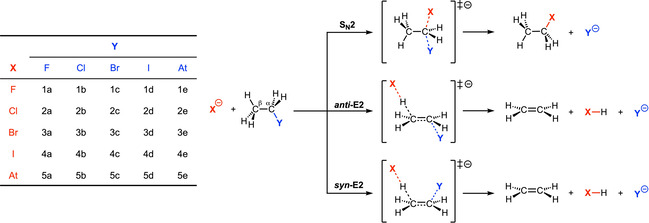
S_N_2 and E2 pathways for reactants X^−^+CH_3_CH_2_Y.

The ability to rationally design chemical reactions is one of the fundamental challenges in chemistry. Unraveling the processes that dictate the course reactants take along a potential energy surface (PES) paves the way to such design and may lead to the discovery of new chemistry. Two prototypical reactions in organic chemistry that feature in many routes in organic synthesis are bimolecular nucleophilic substitution (S_N_2) and base‐induced elimination (E2).[^1^, ^2^] S_N_2 reactions (i.e., nucleophilic attack) are in principle always in competition with E2 reactions (i.e., protophilic attack), which opens the possibility and the necessity to actively tune reactivity toward the desired pathway to maximize the formation of the targeted compound and to avoid unwanted side products (see Scheme 1).

Over the past decades, valuable insights have emerged from experimental^[3]^ and theoretical studies^[4]^ on the trends in S_N_2 and E2 reactivity, as well as the nature of the reactions’ potential energy surfaces.^[2a]^ The direct competition between substitution and elimination pathways of anionic Lewis bases with alkyl substrates is a fundamental problem and the factors that influence this competition in solution have been studied extensively.[^4j^, ^5^, ^6^] Recently, Wu et al.^[7]^ explored the competition between gas phase S_N_2 and E2 pathways for a range of anionic Lewis bases reacting with ethyl chloride. They consolidated our earlier finding that the unfavorably high activation strain, Δ*E* ^≠^_strain_, of the E2 pathway can be overruled by a strongly stabilizing transition state (TS) interaction, Δ*E* ^≠^_int_, eventually leading to a preference for E2 over S_N_2.^[4c]^ Nucleophilicity and leaving group ability in S_N_2 reactions have been related to various properties of X^−^ (the nucleophile) and Y (the leaving group),^[8]^ such as electronegativity, size, polarizability, and others. Nevertheless, the state of the art is to some extent still phenomenological. More recently, it was established that the height of S_N_2 reaction barriers is directly determined by the stability of the nucleophile's (X^−^) highest occupied molecular orbital (HOMO) and by the strength of the substrate's carbon–leaving group bond (C−Y): a higher electron‐donating capability of the X^−^ HOMO or a weaker C−Y bond leads to a lower barrier and vice versa.^[4i]^ The same relations were found by Shaik et al. by using the valence bond (VB) model, who predicted that the height of the S_N_2 barrier depends on the vertical ionization energy of the nucleophile (*I*_X:–_) minus the electron affinity of the C−Y bond (*A*_C−Y_).[^4q^, ^4r^] Where *I*_X:–_ is directly related to the energy of the HOMO and *A*_C−Y_ is dominated by the strength of the C−Y bond.

Herein, we develop, based on quantum chemical analyses, a unified model that provides chemists with the tools to readily understand the duality of Lewis bases, that is their nucleophilic or protophilic character. To this end, we have explored and analyzed the potential energy surfaces along the reaction coordinates of the S_N_2 substitution, *anti*‐E2 elimination (E2‐a), and *syn*‐E2 elimination (E2‐s) reactions of X^−^+C_2_H_5_Y, with X, Y = F, Cl, Br, I, and At, by using relativistic density functional theory (DFT) at ZORA‐OLYP/TZ2P.^[9]^ The C_2_H_5_Y substrate allows us to probe the direct competition between S_N_2 and E2, and our findings can be extended to any substrate where the acidic hydrogen and the leaving group are electronically coupled. In the first place, these explorations provide us with a consistent overview of reactivity trends over a wide range of reactivities and pathways. More importantly, analyses of these consistent reactivity data based on the activation strain model (ASM) of reactivity[^4c^, ^10^] reveal the factors that determine the shape of the potential energy surfaces and hence govern the propensity of the Lewis base to act as a nucleophile or protophile, namely: (i) the “characteristic distortivity” of the substrate, which is associated with a particular reaction mechanism; (ii) the electron‐donating capability of the Lewis base, which enters into an acid–base like interaction with the substrate; and (iii) the strength of the C^α^‐leaving group bond. In the course of our analyses, we develop the concepts of “intrinsic nucleophilicity”, “apparent nucleophilicity”, and “transition state acidity”, which are associated with a particular type of reaction. These concepts will provide chemists with rational design principles that will enable the design of selective synthetic routes to targeted products.

## Results and Discussion

### Main trends in reactivity

**Figure 1:**
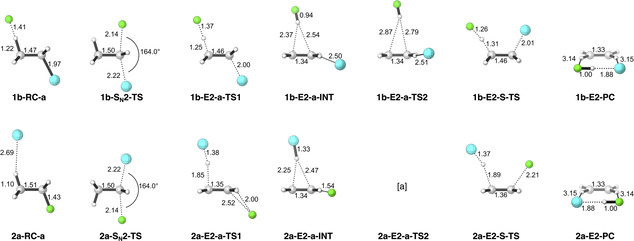
Structures (in Å, deg.) of stationary points in S_N_2, *anti*‐E2, and *syn*‐E2 reactions of F^−^+CH_3_CH_2_Cl (1 b) and Cl^−^+CH_3_CH_2_F (2 a) computed at ZORA‐OLYP/TZ2P. Structures of all model reaction stationary points can be found in the Supporting Information. [a] Nonexistent stationary point, optimization leads directly to the product complex (2 aE2‐PC). Atom colors: carbon (gray), hydrogen (white), fluorine (green), and chlorine (cyan).

The results of our ZORA‐OLYP/TZ2P computations on the S_N_2 and E2 reactions in Scheme 1 are collected in Table 1, in Figure 1–Figure 8, and in the Supporting Information. Table 1 contains the energies of stationary points along the various reaction profiles relative to the energy of the infinitely separated reactants. Structural data of stationary points are shown in Figure 1 for the two representative reactions 1 b and 2 a; full structural data for all stationary points are provided in Figure S1 and Table S1 in the Supporting Information.

**Table 1:** Energies relative to reactants (in kcal mol^−1^) of the stationary points occurring in S_N_2, *anti*‐E2, and *syn*‐E2 reactions of X^−^+C_2_H_5_Y.^[a]^

		Y				
X^−^	species	F (a)	Cl (b)	Br (c)	I (d)	At (e)
F^−^ (1)	RC‐a	−20.0	−23.3	^[b]^	^[b]^	^[b]^
	RC‐s	−15.2	−16.5	^[b]^	^[b]^	^[b]^
	S_N_2‐TS	−4.2	−17.5	^[b]^	^[b]^	^[b]^
	E2‐a‐TS1	−8.0	−23.3	^[b]^	^[b]^	^[b]^
	E2‐a‐INT	−12.5	−37.0	^[b]^	^[b]^	^[b]^
	E2‐a‐TS2	−12.3	−36.7	^[b]^	^[b]^	^[b]^
	E2‐s‐TS	−4.9	−12.6	−15.9	−27.0	−18.7
	E2‐PC	−41.4	−52.2	−57.1	−60.8	−60.5
	S_N_2‐PC	−20.0	−46.6	−55.0	−61.4	−62.3
	S_N_2‐P	0.0	−38.3	−48.4	−56.0	−57.4
	E2‐P	12.9	−25.4	−35.5	−43.1	−44.5
Cl^−^ (2)	RC‐a	−8.4	−9.7	−10.2	−10.9	−10.5
	RC‐s	^[c]^	^[c]^	^[c]^	^[c]^	^[c]^
	S_N_2‐TS	20.8	4.0	−1.6	−5.3	−6.1
	E2‐a‐TS1	36.4	10.7	3.6	−1.3	−2.4
	E2‐a‐INT	32.4	7.5	−0.1	^[d]^	^[d]^
	E2‐a‐TS2	^[d]^	^[d]^	^[d]^	^[d]^	^[d]^
	E2‐s‐TS	39.0	19.6	13.1	8.5	7.1
	E2‐PC	−13.9	−11.8	−14.7	−17.3	−17.1
	S_N_2‐PC	15.1	−9.7	−17.8	−24.0	−24.8
	S_N_2‐P	38.3	0.0	−10.1	−17.7	−19.1
	E2‐P	54.3	16.3	6.2	−1.4	−2.8
Br^−^ (3)	RC‐a	−6.6	−7.7	−8.2	−8.5	−8.3
	RC‐s	^[c]^	^[c]^	^[c]^	^[c]^	^[c]^
	S_N_2‐TS	26.5	8.5	2.9	−1.1	−1.9
	E2‐a‐TS1	46.0	21.4	13.6	7.9	6.9
	E2‐a‐INT	44.4	^[d]^	^[d]^	^[d]^	^[d]^
	E2‐a‐TS2	45.0	^[d]^	^[d]^	^[d]^	^[d]^
	E2‐s‐TS	50.9	27.8	20.7	15.4	14.2
	E2‐PC	−8.8	−4.7	−5.9	−7.3	−7.1
	S_N_2‐PC	^[e]^	−0.1	−8.2	−1.3	−15.2
	S_N_2‐P	48.4	10.1	0.0	−7.6	−9.0
	E2‐P	66.4	28.1	18.0	10.4	9.0
I^−^ (4)	RC‐a	−5.5	−6.4	−6.8	−7.1	−6.8
	RC‐s	^[c]^	^[c]^	^[c]^	^[c]^	^[c]^
	S_N_2‐TS	32.1	12.4	6.5	2.6	1.6
	E2‐a‐TS1	54.5	31.1	23.0	16.9	15.9
	E2‐a‐INT	^[d]^	29.6	21.3	14.8	^[d]^
	E2‐a‐TS2	^[d]^	^[d]^	^[d]^	14.8	^[d]^
	E2‐s‐TS	^[d]^	35.0	27.6	21.9	20.7
	E2‐PC	−4.9	0.3	0.3	−0.2	0.1
	S_N_2‐PC	^[e]^	6.8	−1.0	−7.1	−7.9
	S_N_2‐P	56.0	17.7	7.6	0.0	−1.4
	E2‐P	75.4	37.1	27.0	19.4	18.0
At^−^ (5)	RC‐a	−5.0	−5.8	−6.2	−6.5	−6.2
	RC‐s	^[c]^	^[c]^	^[c]^	^[c]^	^[c]^
	S_N_2‐TS	33.6	13.0	7.0	3.0	2.0
	E2‐a‐TS1	^[d]^	33.9	25.7	19.5	18.4
	E2‐a‐INT	^[d]^	32.4	24.0	17.5	16.5
	E2‐a‐TS2	^[d]^	^[d]^	^[d]^	^[d]^	^[d]^
	E2‐s‐TS	^[d]^	^[d]^	28.9	23.0	21.9
	E2‐PC	−3.2	2.0	1.9	1.5	1.6
	S_N_2‐PC	^[e]^	8.6	0.7	−5.4	−6.2
	S_N_2‐P	57.4	19.1	9.0	1.4	0.0
	E2‐P	77.2	38.9	28.8	21.2	19.8

[a] Computed at ZORA‐OLYP/TZ2P (see Scheme 1 for designation of species). [b] Nonexistent: encounter of reactants induces S_N_2 or E2‐a reaction without barrier. [c] Nonexistent: optimization yields RC‐a. [d] No stationary point obtained due to an extremely shallow PES. [e] Nonexistent: expelled leaving group induced barrierless E2 reaction.

**Figure 2:**
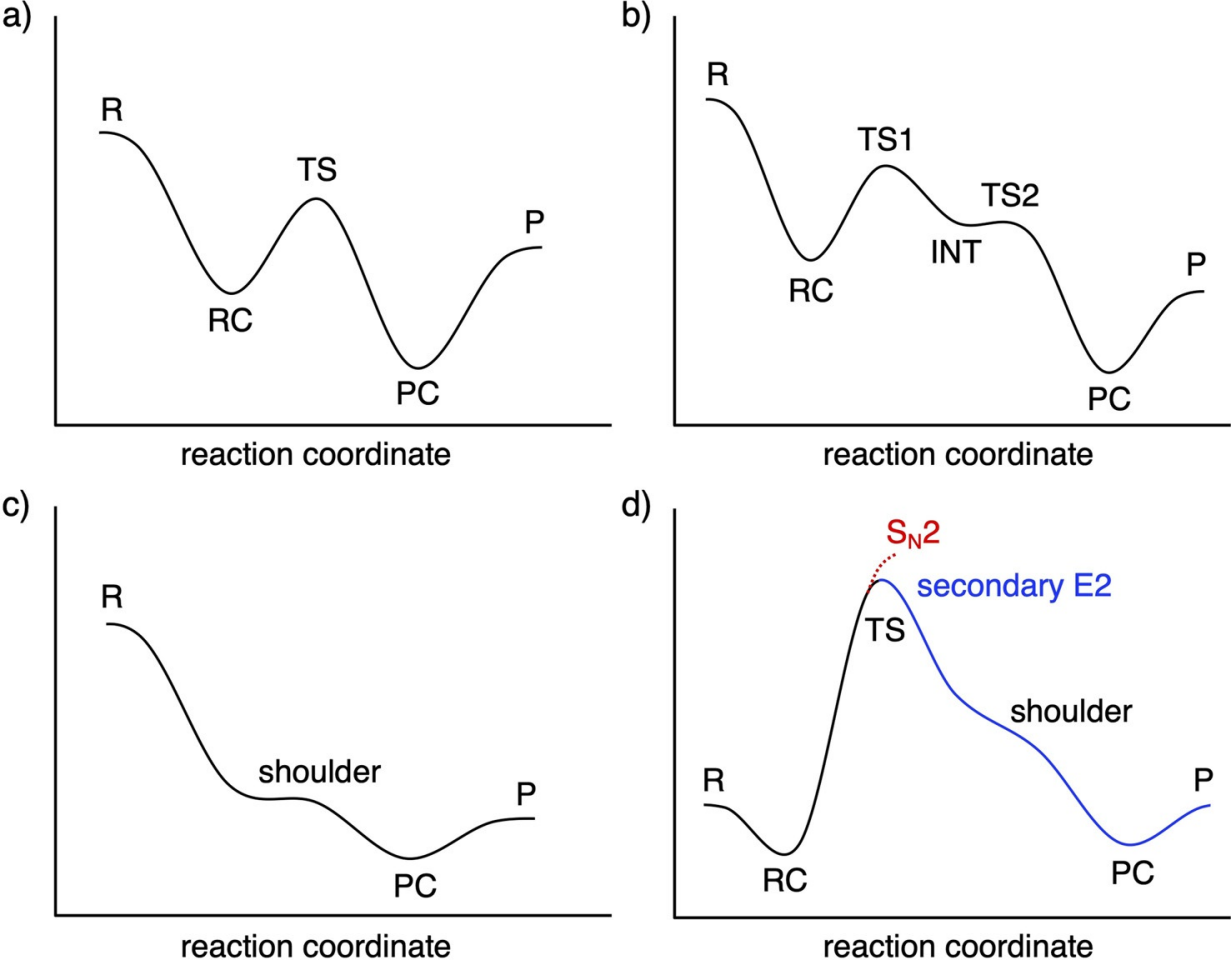
Schematic representation of S_N_2 and E2 potential energy surfaces (PES) computed for the studied X^−^+C_2_H_5_Y systems: (a) The majority of S_N_2 and *syn*‐E2 reactions proceed via a double‐well PES, from reactants (R) and reactant complex (RC) via transition state (TS) to product complex (PC) and products (P). (b) The majority of *anti*‐E2 reactions form at first, via TS1, an intermediate complex (INT), which after a rearrangement, via TS2, yields the same products as *syn*‐E2: an olefin and a leaving group solvated by the conjugated base (C_2_H_4_+YHX^−^). (c) Highly exothermic S_N_2 and *anti*‐E2 reactions may proceed spontaneously, without a central barrier. (d) Highly endothermic S_N_2 pathways have no reverse barrier (red curve); they occur in cases where the leaving group is comparatively basic and spontaneously induces a barrier‐free secondary E2 reaction (blue curve).

In most cases, the S_N_2, *anti*‐E2, and *syn*‐E2 model reactions proceed via a reactant complex (RC) and a transition state (TS) towards a product complex (PC), which may eventually dissociate into products (see Table 1 and Figure 1); exceptions are discussed later on. Schematic representations of such reaction profiles are shown in Figure 2 a for an exothermic reaction. In the case of *anti*‐E2 elimination, the initial transition state (TS1) constitutes the actual elimination process and leads to an intermediate complex (INT) in which the conjugated acid forms an X‐H⋅⋅⋅π complex with the newly formed ethylene and the leaving group Y^−^ hydrogen binds to an ethylene C^α^−H bond (see Figure 1 for selected structures and Figure 2 b for a schematic *anti*‐E2 reaction profile). From here, migration of XH to the leaving group leads, via a second transition state (TS2), to the PC, H_2_C=CH‐H⋅⋅⋅^−^YHX, which, for our model reactions,^[11]^ is identical to that of *syn*‐E2 elimination. In all cases, TS1 is higher in energy than TS2 and, therefore, rate‐determining for the overall *anti*‐E2 pathway. The energetically favored products for both *anti*‐E2 and *syn*‐E2 pathways are C_2_H_4_+YHX^−^, that is, the olefin plus the leaving group, microsolvated by the conjugate acid. A number of clear and general trends in reactivity can be discerned. Reaction barriers always increase as the Lewis base X^−^ becomes less basic, along F^−^, Cl^−^, Br^−^, I^−^, and At^−^ (see Table 1).^[12]^ Note that in the gas phase, it is possible to have negative barriers with respect to the separate reactants, because under these conditions, in many cases, the nucleophile forms an encounter complex (sometimes referred to as an ion‐dipole complex) with the substrate, which is stabilized by both electrostatic and donor–acceptor orbital interactions. Interestingly, reaction barriers rise more rapidly along this series for E2 than for S_N_2 reactions (note that TS1 is rate‐determining for all *anti*‐E2 reactions). This trend can be found for all of the C_2_H_5_Y substrates. As a consequence, the preferred reaction pathway switches from *anti*‐E2, in the cases where F^−^ attacks the substrate, to S_N_2 for the heavier halide anions. For example, along F^−^, Cl^−^, Br^−^, I^−^, and At^−^+C_2_H_5_Cl, the S_N_2 reaction barrier (S_N_2‐TS in Table 1) moderately increases from −17.5 to +4.0, +8.5, +12.4, and +13.0 kcal mol^−1^, respectively, whereas the *anti*‐E2 barrier (E2‐a‐TS1 in Table 1) rises more steeply from −23.3 to +10.7, +21.4, +31.1, and +33.9 kcal mol^−1^, respectively. Thus, although *anti*‐E2 prevails for the more basic halide F^−^, with a reaction barrier that is 5.8 kcal mol^−1^ *lower* than the S_N_2 pathway, the S_N_2 pathway dictates for all heavier, less basic, halides, with an *anti*‐E2 barrier for At^−^ that is 20.9 kcal mol^−1^ *higher* than the S_N_2 pathway. This is in line with the work of Shaik et al., who showed, with the use of valence bond (VB) theory, that strong Lewis bases prefer the E2 pathway.^[13]^ The *syn*‐E2 pathway is in all cases less reactive than *anti*‐E2.

Our S_N_2 barriers for X^−^+C_2_H_5_Y are consistently a few kcal mol^−1^ higher than the corresponding barriers for X^−^+CH_3_Y obtained at the same level of theory in an earlier study.^[4i]^ For example, along F^−^, Cl^−^, Br^−^, and I^−^+CH_3_Cl, the S_N_2 barrier increases comparatively moderately from −19.2 to −0.2, +4.1, and +7.9 kcal mol^−1^.^[4i]^ This is consistent with the slight increase of steric hindrance in the S_N_2 reactions of C_2_H_5_X compared with those of CH_3_X.^[14]^ On the other hand, reaction barriers decrease for both S_N_2 and *anti*‐E2 pathways as the leaving group Y in the substrate C_2_H_5_Y varies along F, Cl, Br, I, and At. Thus, along Cl^−^+C_2_H_5_F, C_2_H_5_Cl, C_2_H_5_Br, C_2_H_5_I, and C_2_H_5_At, the S_N_2 barrier (S_N_2‐TS in Table 1) decreases from +20.8 to +4.0, −1.6, −5.3, and −6.1 kcal mol^−1^ whereas the *anti*‐E2 barrier (E2‐TS1 in Table 1) goes from +36.4 down to +10.7, +3.6, −1.3, and −2.4 kcal mol^−1^.

Our computations show that less basic halides, that is, those with a lower proton affinity, are both worse nucleophiles and worse protophiles, in the sense that they lead to higher barriers for substitution (nucleophilic attack) as well as for elimination (protophilic attack) reactions along the series F^−^<Cl^−^<Br^−^<I^−^<At^−^. Thus, if there were no competing E2 channels, for example, in the aforementioned reaction systems X^−^+CH_3_Y,^[4i]^ a stronger Lewis base is a better nucleophile. This is what we designate as “intrinsic nucleophilicity”. However, our computations also show that the lowering of reaction barriers for the protophilic attack benefits more from increasing the basicity than that for the nucleophilic attack. Thus, if the basicity becomes strong enough, the protophilic character of X^−^ prevails. In this situation of mechanistic competition, we speak about the “apparent nucleophilicity”. Note that weaker Lewis bases proceed with a reduced intrinsic nucleophilicity (i.e., higher S_N_2 barrier) but an enhanced apparent nucleophilicity (i.e., more favorable S_N_2 barrier compared with E2 barrier). The origin of these trends is analyzed and explained later on, on the basis of the activation strain model (ASM) of reactivity[^4c^, ^10^] and quantitative molecular orbital (MO) theory.^[15]^

### Special features of particular reactions

The prior discussed trends in S_N_2 versus E2 reactivity hold for all reaction systems. But the precise shape of the PES differs in a few instances to the extent that the process becomes spontaneous, the reverse barrier disappears, or the product complex becomes labile and leads to a spontaneous follow‐up reaction.

In the case of the rather exothermic reactions that occur between F^−^ and C_2_H_5_Br, C_2_H_5_I, or C_2_H_5_At, the barrier for the *anti*‐E2 pathway disappears and F^−^ spontaneously abstracts a β‐proton from C_2_H_5_X (X = Br, I, At) to form the product complex E2‐a‐PC, C_2_H_4_⋅⋅⋅^−^YHX, without the occurrence of a stable reactant complex or transition state. The latter has become a shoulder on the PES along the reaction coordinate, as schematically depicted in Figure 2 c. The barrier for the S_N_2 reaction has also disappeared for these reactants, which is in line with our previously obtained results for the S_N_2 reactions F^−^+CH_3_Br and CH_3_I.^[4i]^ However, the steepest descent path upon the encounter of the F^−^+C_2_H_5_X reactants leads into the *anti*‐E2 and not the S_N_2 channel.

**Scheme 2:**
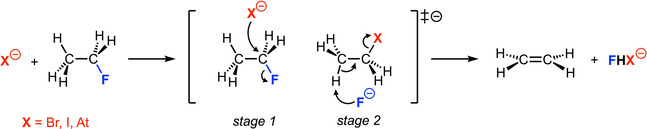
Multi‐step reaction found for highly endothermic nucleophilic substitutions.

The highly endothermic nucleophilic substitutions between Br^−^, I^−^, and At^−^+C_2_H_5_F have, by symmetry, no reverse barrier (see Figure 2 d, red dotted curve). Interestingly, when following the three forward S_N_2 processes, we nevertheless do find saddle‐points at 26.5, 32.1, and 33.6 kcal mol^−1^, respectively (listed in Table 1, as S_N_2‐TS). This transition state is achieved after the actual substitution stage, as the reaction systems begin to deviate from the actual S_N_2 path. What happens is that the emerging leaving group, Y^−^=F^−^, is a relatively strong Lewis base, which induces a barrier‐free E2 elimination from the comparatively reactive C_2_H_5_X molecule (X = Br, I, At) formed in the S_N_2 reaction (Scheme 2). This is schematically depicted in Figure 2 d, blue curve. Successive S_N_2+E2 multi‐step reactions have also been observed by using mass spectroscopic techniques in other reaction systems.^[16]^ Eventually, the same E2‐a‐P product, C_2_H_4_⋅⋅⋅FHX^−^, is formed as in a direct E2 reaction between the original reactants. For example, in the case of Br^−^+C_2_H_5_F, the S_N_2 pathway, with a barrier of only 26.5 kcal mol^−1^, dominates the direct *anti*‐E2 reaction, with a barrier of 46.0 kcal mol^−1^. Yet, also the S_N_2 pathway leads, via a concerted S_N_2+E2 mechanism, to the formation of C_2_H_4_ and FHBr^−^ and not C_2_H_5_Br and F^−^.

### Activation strain analyses

**Figure 3:**
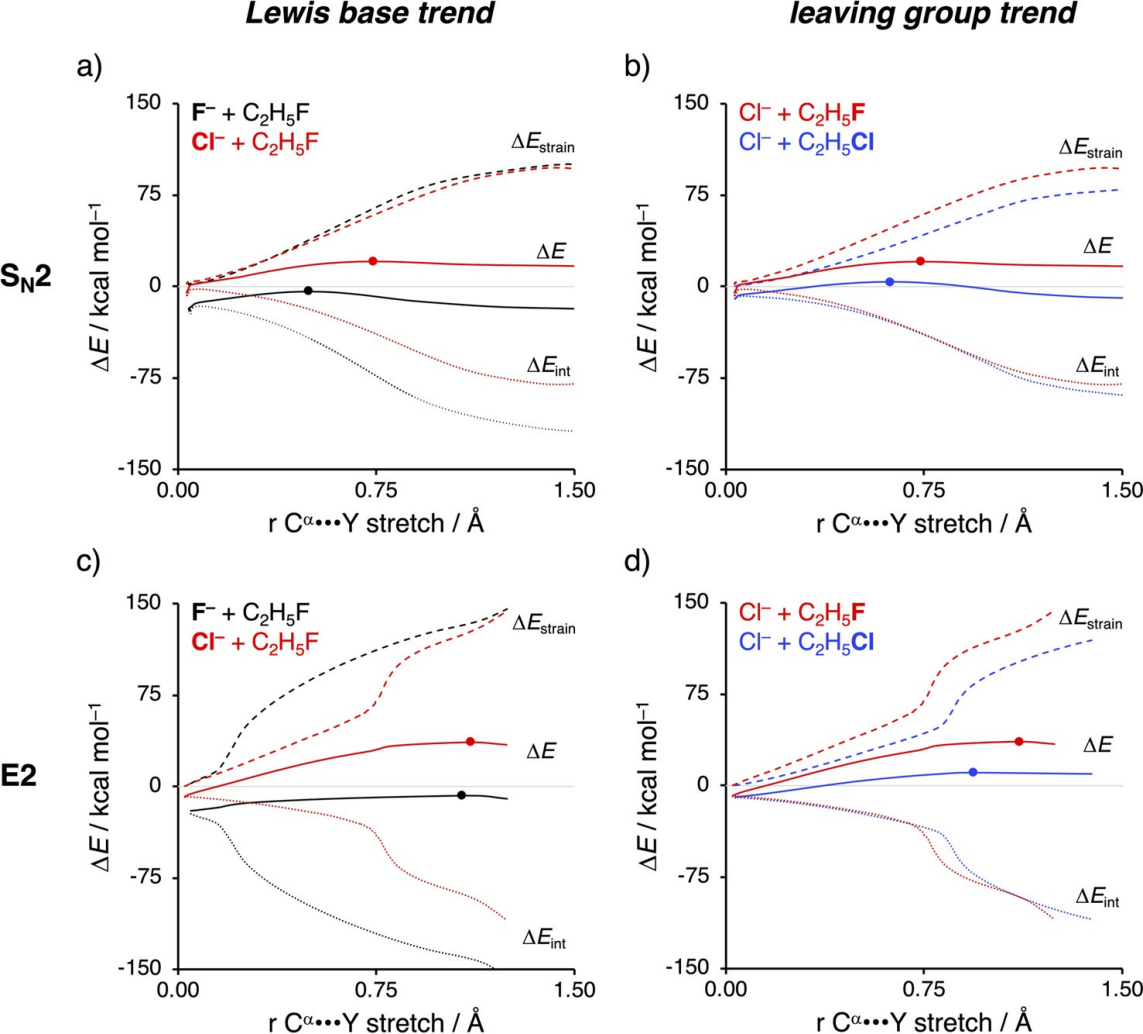
Activation strain analysis of the S_N_2 and *anti*‐E2 reactions of X^−^+C_2_H_5_Y with X, Y = F, Cl. The left column (a, c) shows how variation of the Lewis base influences the PES, whereas the right column (b, d) shows the effect of leaving group variation. Solid lines correspond to the PES, dashed lines to the strain energy, and dotted curves to the interaction energy. Transition states are indicated with dots. Computed at ZORA‐OLYP/TZ2P.

**Figure 4:**
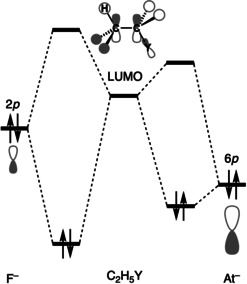
Schematic orbital interaction diagram between the filled n *p* HOMO of X^−^ (F^−^: left; At^−^: right) and the LUMO of C_2_H_5_Y (middle). Note that the substrate LUMO has σ* antibonding character in both the C^α^−Y and C^β^−H bonds.

**Figure 5:**
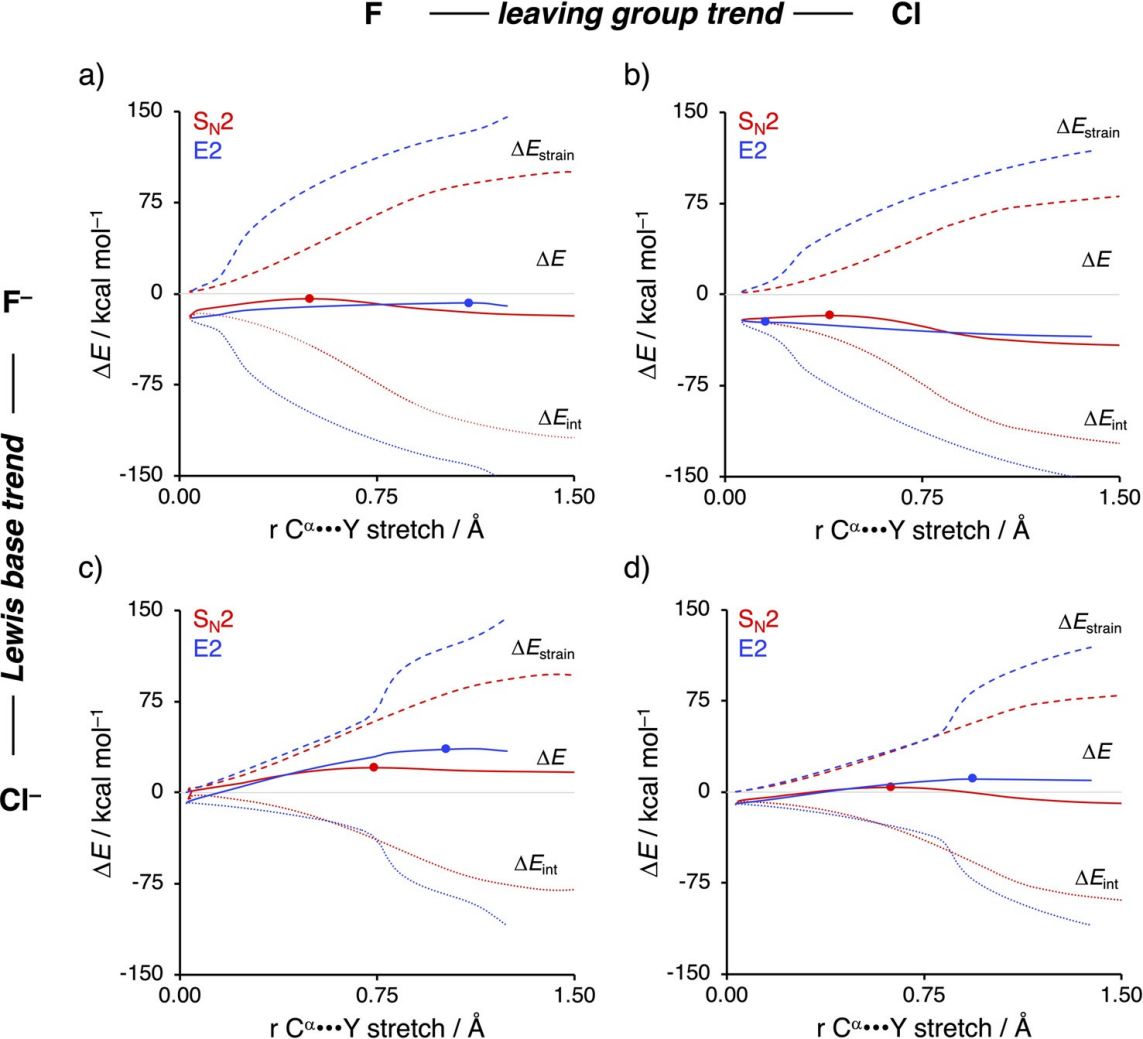
Activation strain analysis of the differences between the PESs of S_N_2 (red) and *anti*‐E2 (blue) reactions of X^−^+C_2_H_5_Y with X, Y = F, Cl. Trends down columns (a→c or b→d) show how variation of the Lewis base influences the competition, whereas trends along rows (a→b or c→d) show the effect of leaving group variation. Solid lines correspond to the PES, dashed lines to the strain energy, and dotted lines to the interaction energy. Transition states are indicated with dots. Computed at ZORA‐OLYP/TZ2P.

The results of our activation strain analysis (ASA)[^4c^, ^10^] for the representative S_N_2 and *anti*‐E2 reactions of X^−^ and C_2_H_5_Y (X, Y = F, Cl) are collected in Figure 3 and Figure 5 (see Figure S2 in the Supporting Information for all data). The activation strain model involves the decomposition of the electronic energy (Δ*E*) into two distinct energy terms, namely, the strain energy (Δ*E*_strain_) and the interaction energy (Δ*E*_int_). The strain energy results from the deformation of the individual reactants and the interaction energy between the deformed reactants along the reaction coordinate, defined, in this case, as the stretch of the α‐carbon–leaving group (C^α^−Y) bond. This critical reaction coordinate undergoes a well‐defined change during the reaction from the reactant complex via the transition state to the product and is shown to be a valid reaction coordinate for studying substitution reactions.[^4i^, ^17^] Note that the *syn*‐E2 pathway always goes with a higher reaction barrier than the *anti*‐E2 pathway and, therefore, is excluded from this analysis. In Figure 3, we show how the nature of the Lewis base X^−^ (left column) and the leaving group Y (right column) influences the decomposition of the potential energy surface (PES) along the reaction coordinate (ζ), cf. Eq. (1), for the S_N_2 reaction (upper row) and *anti*‐E2 reaction (lower row). The solid curves represent the PES (Δ*E*), whereas the dashed and dotted curves represent the strain (Δ*E*_strain_) and interaction (Δ*E*_int_) energy, respectively. Panels (a) and (c) compare curves of F^−^+C_2_H_5_F (black) and Cl^−^+C_2_H_5_F (red) for S_N_2 and *anti*‐E2 reactions, respectively, whereas panels (b) and (d) compare curves of Cl^−^+C_2_H_5_F (red) and Cl^−^+C_2_H_5_Cl (blue) for S_N_2 and *anti*‐E2 reactions, respectively. Note that the left and right columns share reaction 1 a, that is, Cl^−^+C_2_H_5_F. This series is representative for the observed effects induced by Lewis base and/or leaving group variations along the various model reactions. Figure 3 a indicates that, in the S_N_2 reaction, a stronger nucleophile enhances, in agreement with its increased intrinsic nucleophilicity, the stabilizing interaction energy over the entire course of the reaction, whereas the strain energy is minimally affected. The reason for this more stabilizing interaction energy is the stability of the X^−^ n *p* atomic orbital (AO), which decreases along At^−^, I^−^, Br^−^, Cl^−^, and F^−^ and reduces the corresponding HOMO–LUMO energy gap with the substrate (Figure 4).^[18]^ This effect can be explained by the size of the AOs of the nucleophile. F^−^ has a less stable HOMO owing to the compactness of fluorine AOs, which experience more destabilizing coulombic repulsion between the electrons compared with the heavier and larger halides. A better leaving group, on the other hand, results in a weaker carbon–leaving group bond, that is, lower carbon–leaving group bond enthalpy,^[19]^ which manifests in less destabilizing strain energy, whereas the interaction energy is hardly affected by varying the leaving group (Figure 3 b).

Similar trends are observed for the E2 reaction. In Figure 3 c, the variation of the protophile, the situation is slightly more complicated as a stronger protophile results in an earlier proton abstraction, that is, an earlier jump in interaction and strain energy, along the reaction coordinate. The interaction energy is largely influenced by the nature of the protophile, because a stronger protophile, due to its enhanced intrinsic nucleophilicity, results in a more stabilizing interaction and, therefore, a lower transition barrier (see above). Furthermore, the nature of the protophile affects the strain energy by abstracting the proton at different moments along the reaction coordinate, which can be seen as the different positions of the sudden jump in strain energy. The stronger the base, the earlier it abstracts the proton. Note that the strain energy around the reactant and product complexes (i.e., start and end of the activation strain diagram) are nearly consistent and hence not influenced by the nature of the protophile. In line with the S_N_2 systems, a better leaving group reduces the strain curves, as a result of the prior discussed weaker carbon–leaving group bond, whereas the stabilizing interaction energy remains nearly unchanged (Figure 3 d). Thus, a better Lewis base or leaving group results in both a lower S_N_2 and E2 reaction barrier.

To directly analyze and compare the S_N_2 and E2 pathways, Figure 5 shows four panels displaying the S_N_2 and E2 pathways of the model reaction: F^−^+C_2_H_5_F (1 a), F^−^+C_2_H_5_Cl (1 b), Cl^−^+C_2_H_5_F (2 a), and Cl^−^+C_2_H_5_Cl (2 b). Going down a column, we vary the Lewis base, and along a row, we change the nature of the leaving group. Note that, for all reactions, the strain and interaction energy curves for the E2 reaction display a profound difference compared to the S_N_2 analog. As mentioned above, a sudden jump in strain and interaction energy is observed during the E2 reaction. This jump can be attributed to the proton abstraction by the Lewis base, which, in E2 reactions, acts as a protophile. The deprotonation of the substrate by the protophile requires a large deformation in the geometry of the substrate but also results in a more stabilizing interaction (see below).

**Figure 6:**
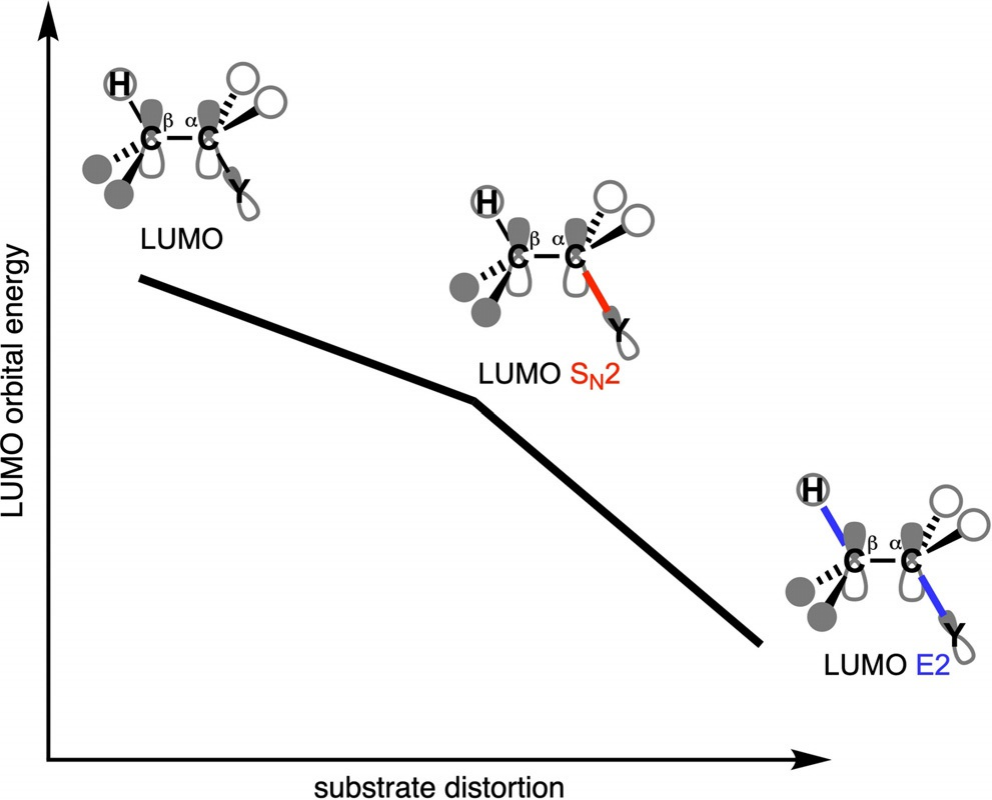
Schematic representation of how the LUMO energy is affected by increasingly distorting the substrate (C_2_H_5_Y) from its equilibrium geometry to the S_N_2, and to the E2 pathway.

The S_N_2 pathway intrinsically has a less destabilizing strain energy than the E2 analog, because along the former reaction pathway only one bond (C^α^−Y) is being broken, while for the latter two bonds are being broken (C^α^−Y and C^β^−H). Thus, the distortion, characteristic for the S_N_2 pathway, is inherently lower than the E2 pathway. At the same time, the “characteristic distortivity” for both pathways also has direct implications on the electronic structure of the substrate. The LUMO of the substrate has antibonding character in the C^α^−Y and C^β^−H bonds. The deformation along the S_N_2 pathway (elongation of C^α^−Y) reduces the antibonding overlap for C^α^−Y, which, in turn, stabilizes the LUMO (see Figure 6). For the E2 reaction, this effect is more pronounced as the antibonding overlap of both the C^α^−Y and C^β^−H bonds are being reduced. For the S_N_2 pathway, this results in an intrinsically larger HOMO–LUMO gap than for the E2 pathway, and therefore a significantly less stabilizing interaction energy between the Lewis base and the substrate, regardless of the Lewis base.

Our activation strain analysis reveals that, similar to the strain energy, the interaction energy may also be translated into a simple concept, that is, it corresponds directly to the strength of the Lewis acid or base.[^1^, ^20^] A more basic Lewis base (higher‐energy HOMO) interacts more strongly. In addition, a more acidic substrate (lower‐energy LUMO) also interacts more strongly. Consequently, we propose the novel concept of effective acidity of the deformed substrate in the transition state, or “transition state acidity”. For an E2 pathway, the substrate in the transition state is more acidic (lower‐energy LUMO), whereas in an S_N_2 pathway it is less acidic (higher‐energy LUMO). As a result, the E2 pathways will always dominate the S_N_2 pathway in the limit of a strong interaction (more basic Lewis base), which we have observed for the reactions where X^−^=F^−^.

Changing the Lewis base from X^−^=F^−^ to X^−^=Cl^−^ has a profound effect on the preferred reaction pathway, shifting the preference from E2 for F^−^ (Figure 5 a and b) to S_N_2 for Cl^−^ (Figure 5 c and d). As previously discussed, when going from F^−^ to Cl^−^ the basicity is reduced, which manifests in a less stabilizing interaction energy for both the S_N_2 and E2 reaction pathways. This enhances the apparent nucleophilicity, because the S_N_2 barrier becomes more favorable compared with the E2 barrier. The weaker Lewis base Cl^−^ has a lower‐energy HOMO (Figure 4), resulting in a larger HOMO–LUMO gap and hence a weaker interaction with the substrate. Due to this weaker interaction, Cl^−^ is unable to overcome the highly destabilizing characteristic distortivity that inextricably accompanies the E2 reaction.

**Figure 7:**
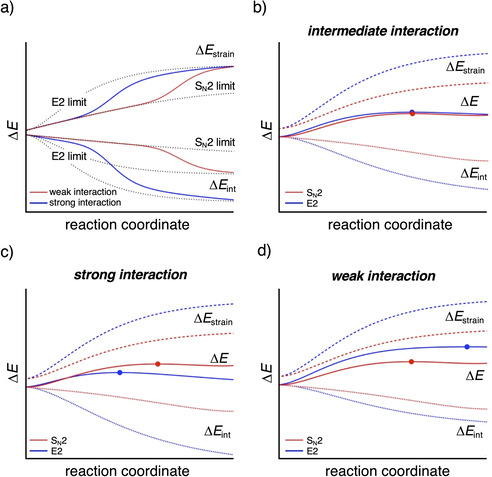
(a) Extrapolated strain and interaction curves to a simplified S_N_2 and E2 limit. Altering the strength of the acid–base interaction from (b) intermediate, to (c) strong to (d) weak.

On the other hand, substituting Y for a better leaving group, by going from Y = F to Y = Cl, reduces the strain curves for the S_N_2 and E2 pathway to a similar extent, making the strain a less important factor, whereas the interaction curves, which are always in favor of E2, remain essentially constant for both pathways. As predicted by our model, this has the effect of reducing the apparent nucleophilicity. Thus, the preference for the E2 pathway is further enhanced (e.g., from F^−^+C_2_H_5_F to F^−^+C_2_H_5_Cl) or the preference for the S_N_2 pathway is reduced (e.g., from Cl^−^+C_2_H_5_F to Cl^−^+C_2_H_5_Cl); see also Table 1 and Figure 5. At last, we were able to extrapolate the strain and interaction curves of our model reactions to a simplified S_N_2 and E2 limit (see Figure 7 a). This plot clearly displays the interaction of the Lewis base with the acidic substrate to be the dominant effect that determines the propensity towards the S_N_2 or E2 reaction pathway.

Our herein presented model also explains the effect of solvation on the S_N_2 versus E2 competition. Solvation stabilizes the lone‐pair electrons of a Lewis base and, thus, lowers the HOMO of X^−^ and reduces its electron‐donating capability or basicity. As a response, the acid–base, that is, HOMO–LUMO, interaction between the Lewis base and substrate goes from a stronger interaction, for example, in the case of F^−^ (Figure 7 c), to a weaker interaction (Figure 7 d) and, hence, changes the preferred reaction pathway from E2 in the gas phase to S_N_2 in solution.[^3m^, ^4b^, ^4j^, ^6a^, ^6e^] In addition, also for weaker Lewis bases (X^−^=Cl^−^, Br^−^, I^−^, At^−^), solvation will enhance the apparent nucleophilicity as it increases the E2 reaction barrier to a larger extent than the S_N_2 reaction barrier. These effects will be more pronounced when the polarity of the solvent increases.^[21]^

### Evaluating the generality of the model

Next, we seek to test our proposed general model and have, therefore, studied the S_N_2/E2 competition of the following three, commonly used, Lewis bases H_3_CHN^−^, H_3_CO^−^, and H_3_CS^−^ with C_2_H_5_Cl.[^4b^, ^7^, ^22^] As previously discussed, strong Lewis bases will have a more favorable interaction with the substrate than weak Lewis bases and, therefore, the former will be able to overcome the characteristic high distortivity accompanied with the E2 reaction. Thus, based on the strength of the Lewis base, that is, the stability of the HOMO, one can predict the preferred reaction pathway. The energy of the HOMO of the three Lewis bases decreases from H_3_CHN^−^ (*ϵ*_HOMO_=3.3 eV), to H_3_CO^−^ (*ϵ*_HOMO_ = 2.4 eV), to H_3_CS^−^ (*ϵ*_HOMO_ = 1.7 eV), which indicates that the Lewis base becomes increasingly weaker. This implies that the strong Lewis base H_3_CHN^−^ will be prone to undergo an E2 reaction and that the intrinsic nucleophilicity reduces along the series from H_3_CHN^−^, to H_3_CO^−^, to H_3_CS^−^.

Table 2 displays the energies of the stationary points of the S_N_2 and E2 reaction between H_3_CX^−^ (X = HN, O, S) and C_2_H_5_Cl. As predicted, based on the stability of the HOMO of the Lewis base, H_3_CHN^−^ is the most reactive Lewis base, to the extent that both the S_N_2 and E2 reactions are barrierless. We note that the S_N_2 reaction occurs with a TS‐like structure at −13.7 kcal mol^−1^ but this is a shoulder on the reactions’ potential energy surface, as shown in Figure 2 c, not a saddle point. Interestingly, even though H_3_CO^−^ is a moderate Lewis base, it is strong enough to result in a lower reaction barrier for the E2 reaction compared to the S_N_2 reaction, −12.1 and −9.2 kcal mol^−1^, respectively. Contrarily, the weakest Lewis base of the series, H_3_CS^−^, undergoes, not unexpectedly, an S_N_2 reaction, with a barrier that is 3 kcal mol^−1^ lower than the E2 reaction. Thus, changing the Lewis base from H_3_CHN^−^ to H_3_CO^−^ to H_3_CS^−^ reduces the intrinsic nucleophilicity, as the S_N_2 reaction barrier steadily increases, but enhances the apparent nucleophilicity, because the S_N_2 reaction barrier becomes consistently more favorable compared with the E2 barrier.

**Table 2:** Energies relative to reactants (in kcal mol^−1^) of the stationary points occurring in S_N_2 and E2 of H_3_CX^−^+C_2_H_5_Cl (X = HN, O, S).^[a]^

	H_3_CX^−^		
	H_3_CHN^−^	H_3_CO^−^	H_3_CS^−^
RC	^[b]^	−13.1	−8.7
S_N_2‐TS	^[b,c]^	−9.2	−1.8
E2‐TS	^[b]^	−12.1	1.5
S_N_2‐PC	−77.4	−47.3	−34.1
E2‐PC	−66.4	−48.3	−21.9
S_N_2‐P	−68.0	−43.0	−25.1
E2‐P	−54.0	−29.7	−6.8

[a] Computed at ZORA‐OLYP/TZ2P. [b] Nonexistent: encounter of reactants induces S_N_2 and E2 reactions without barrier. [c] An IRC analyses reveals a shoulder along the S_N_2 potential energy surface at −13.7 kcal mol^−1^, which is characterized by forming the new C^α^−X bond and breaking the old C^α^−Y.

**Figure 8:**
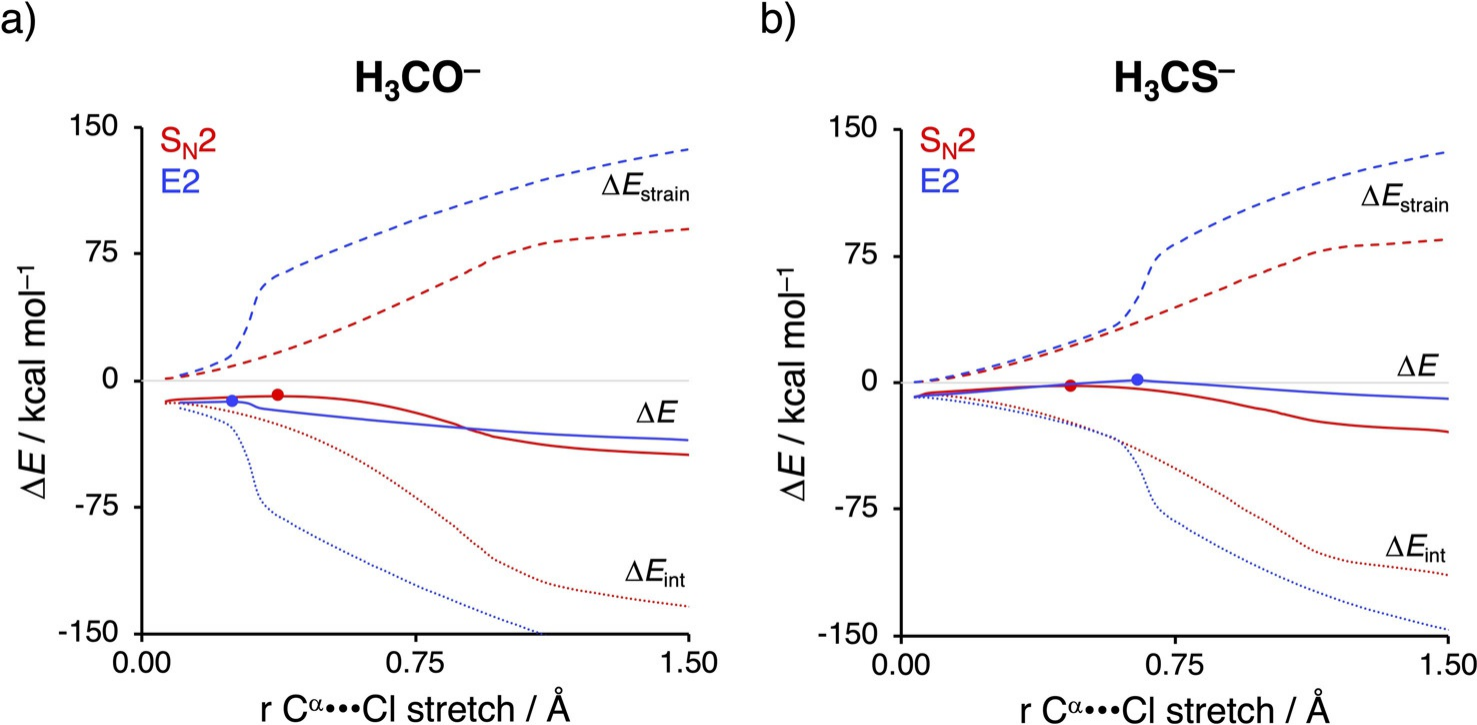
Activation strain diagram of the difference in the PESs of the S_N_2 (red) and E2 (blue) reactions of (a) H_3_CO^−^ and (b) H_3_CS^−^ with C_2_H_5_Cl. Solid lines correspond to the PES, dashed lines to the strain energy, and dotted lines to the interaction energy. Transition states are indicated with dots. Computed at ZORA‐OLYP/TZ2P.

At last, we applied the activation strain model (ASM) of reactivity to examine if the behavior of the Lewis base, that is, nucleophilic or protophilic, is indeed determined by the Lewis acid–base‐like interaction between the Lewis base and the substrate. In Figure 8, we focus on the S_N_2/E2 competition of H_3_CO^−^ and H_3_CS^−^, which prefer an E2 and S_N_2 reaction, respectively. It can clearly be seen that the more basic Lewis base H_3_CO^−^ interacts strongly with the more acidic E2 transition state, which, in turn, manifests in a more stabilizing interaction energy (Figure 8 a). As a result, H_3_CO^−^ is able to overcome the highly destabilizing characteristic distortivity along the E2 pathway and hence making H_3_CO^−^ a protophile. On the other hand, H_3_CS^−^ is a weaker Lewis base and, for that reason, has a less stabilizing Lewis acid–base‐like interaction with C_2_H_5_Cl, resulting in reaction barriers that are determined by the strain energy (Figure 8 b). As the S_N_2 reaction occurs with less destabilizing strain energy, i.e., a lower characteristic distortivity, than the E2 pathway, H_3_CO^−^ will act as a nucleophile following the S_N_2 reaction. The herein presented results show that our proposed model is indeed general and can be used to elucidate the S_N_2/E2 competition of a plethora of Lewis bases.

## Conclusion

Bimolecular nucleophilic substitution (S_N_2; nucleophilic attack) and base‐induced elimination (E2; protophilic attack) reactions are both accelerated when the electron‐donating capability of the Lewis base increases, but the E2 pathway benefits more and therefore is favored in the case of stronger Lewis bases. Solvation, in general, stabilizes the HOMO, decreasing the electron‐donating capability of the Lewis base and thus reduces the preference for E2 or enhances the preference for S_N_2 (enhanced apparent nucleophilicity), even though the barrier of the latter is also raised (reduced intrinsic nucleophilicity). These insights emerge from a detailed and consistent quantum chemical exploration of a vast range of archetypal model systems X^−^+C_2_H_5_Y (X, Y = F, Cl, Br, I, At) displaying a wide range in reactivity and pathways.

We highlight the main factors determining the shape of the potential energy surface, and hence the propensity of the Lewis base to act as a nucleophile or protophile, to be the structural deformation of the substrate during the course of the reaction in combination with the nature of the Lewis base and the nature of the leaving group. Each pathway is associated with a characteristic distortivity: high and associated with a more destabilizing strain for the E2 pathway, in which two bonds are broken (C^α^−Y, C^β^−H), versus, low and associated with a less destabilizing strain for the S_N_2 pathway, in which only one bond is broken (C^α^−Y). At the same time, the LUMO of the substrate is C^α^−Y and C^β^−H antibonding and therefore assumes a lower orbital energy along the more distortive E2 pathway, rendering effectively a higher electron‐accepting capability. We refer to this circumstance as the “transition state acidity” of the substrate, which is stronger for E2 than S_N_2.

Thus, the Lewis acid–base‐like interaction between the Lewis base and the substrate in the transition state determines the outcome of the competition: (i) in a regime of weak interaction, that is, if the Lewis base is weak, the strain determines the barrier and this factor is always more favorable, i.e., less destabilizing, for the less distortive pathway, S_N_2; (ii) in a regime of strong interaction, that is, if the Lewis base is strong, the interaction overrules the strain and determines the barrier, and this factor is always more favorable, i.e., more stabilizing, for the more distortive pathway, E2. These findings show that the nucleophilic or protophilic behavior of a Lewis base towards a Lewis‐acidic substrate is fundamentally co‐determined by the latter.

The introduced concepts of “characteristic distortivity” and “transition state acidity”, together with the distinction between apparent and intrinsic nucleophilicity, provide a vital, qualitative approach for understanding organic reactions in the framework of both MO theory and Lewis’ theory of acids and bases.[^15^, ^20^] This approach rationalizes in a physically sound and intuitive manner why strong Lewis bases prefer the protophilic pathway, whereas weak Lewis bases behave as nucleophiles in S_N_2 reactions, and why (stronger) solvation pushes the mechanistic competition from E2 towards S_N_2. The insights provided herein elucidate a plethora of experimental findings and can serve as powerful tools for a more rational design of synthetic routes. We envisage that the scope of our findings extends well beyond the competition between nucleophilic and protophilic reactivity.

## Conflict of interest

The authors declare no conflict of interest.

## Supporting information

As a service to our authors and readers, this journal provides supporting information supplied by the authors. Such materials are peer reviewed and may be re‐organized for online delivery, but are not copy‐edited or typeset. Technical support issues arising from supporting information (other than missing files) should be addressed to the authors.

Supplementary
