# Supplementary material for: A Unified Framework for Understanding Nucleophilicity and Protophilicity in the SN2/E2 Competition
Source: Chemistry. 2020 Oct 22;26(67):15538–48. doi: 10.1002/chem.202003831 (PMC7756690; doi:10.1002/chem.202003831)
Supplement: Supplementary file 1 — Supplementary [file CHEM-26-15538-s001.pdf]

Chemistry–A European Journal

Supporting Information

A Unified Framework for Understanding Nucleophilicity and Protophilicity in the S_N2/E2 Competition

Pascal Vermeeren+,[a] Thomas Hansen+,[a, b] Paul Jansen,[a, c] Marcel Swart,[d, e] Trevor A. Hamlin,*[a] and F. Matthias Bickelhaupt*[a, f]

Contents

Computational details

Figure S1. Structures of stationary points in S_N2, *anti*-E2 and *syn*-E2 reactions of X^- + CH_3CH_2Y, computed at ZORA-OLYP/TZ2P.

Figure S2. Activation strain analysis of S_N2 and *anti*-E2 reaction profiles along the reaction coordinate projected onto the C–Y stretch, computed at ZORA-OLYP/TZ2P.

Table S1. Cartesian coordinates of all $[C_2H_5XY]^-$ intermediates and transition states, computed at ZORA-OLYP/TZ2P.

Table S2. Cartesian coordinates of all reactants, CH_3–CH_2Y rotational transition states and products, computed at ZORA-OLYP/TZ2P.

Table S3. Cartesian coordinates of H_3CHN^-, H_3CO^-, or H_3CS^-, + $C_2H_5Cl^-$ intermediates and transition states, computed at ZORA-OLYP/TZ2P.

Computational details

All calculations have been carried out using the Amsterdam Density Functional (ADF) and QUILD programs.[1] Equilibrium and transition state geometries were fully optimized at ZORA-OLYP/TZ2P.[2] In previous benchmark studies, we have shown that OLYP reproduces S_N2 barriers from highly correlated *ab initio* within only a few kcal mol^{-1}.[3] Relativistic effects were accounted for by using the zeroth-order regular approximation (ZORA).[2e] The basis set used, denoted TZ2P, is of triple-ζ quality for all atoms and has been improved by two sets of polarization functions.[2d] All stationary points were confirmed by vibrational analysis: for equilibrium structures, all normal modes have real frequencies, whereas transition states have one normal mode with an imaginary frequency. The potential energy surfaces of the studied substitution and elimination reactions were obtained by performing intrinsic reaction coordinate (IRC) calculations,[4] which, in turn, were analyzed using the PyFrag program.[5]

---

[1] a) G. te Velde, F. M. Bickelhaupt, E. J. Baerends, C. Fonseca Guerra, S. J. A. van Gisbergen, J. G. Snijders, T. Ziegler, *J. Comput. Chem.* **2001**, *22*, 931; b) C. Fonseca Guerra, J. G. Snijders, G. te Velde, E. J. Baerends, *Theor. Chem. Acc.* **1998**, *99*, 391; c) ADF 2010.01, SCM Theoretical Chemistry, Vrije Universiteit : Amsterdam (The Netherlands), **2010** http://www.scm.com; d) M.; Swart, F. M. Bickelhaupt, *J. Comput. Chem.* **2008**, *29*, 724.

[2] a) N. C. Handy, A. J. Cohen, *Mol. Phys.* **2001**, 99, 403. (b) C. Lee, W. Yang, R. G. Parr, *Phys. Rev. B: Condens. Matter Mater. Phys.* **1988**, *37*, 785; c) J. Baker, P. Pulay, *J. Chem. Phys.* **2002**, 117, 1441; d) E. van Lenthe, E. J. Baerends, *J. Comput. Chem.* **2003**, *24*, 1142; e) E. van Lenthe, E. J. Baerends, J. G. Snijders, *J. Chem. Phys.* **1994**, 101, 9783.

[3] a) M. Swart, M. Solà, F. M. Bickelhaupt, *J. Chem. Theory Comput.* **2010**, 6, 3145; b) M. Swart, A. W. Ehlers, K. Lammertsma, *Mol. Phys.* **2004**, 102, 2467; c) X.; Xu, W. A. Goddard III, *J. Phys. Chem. A* **2004**, 108, 8495; d) J. M.; Gonzales, W. D.; Allen, H. F., Schaefer III, *J. Phys. Chem. A* **2005**, 109, 10613.

[4] a) K. Fukui, *Acc. Chem. Res.* **1981**, *14*, 363; b) L. Deng, T. Ziegler, L. A. Fan, *J. Chem. Phys.* **1993**, *99*, 3823; c) L. Deng, T. Ziegler, *Int. J. Quantum Chem.* **1994**, *52*, 731.

[5] X. Sun, T. M. Soini, J. Poater, T. A. Hamlin, F. M. Bickelhaupt, *J. Comp. Chem.* **2019**, *40*, 2227.

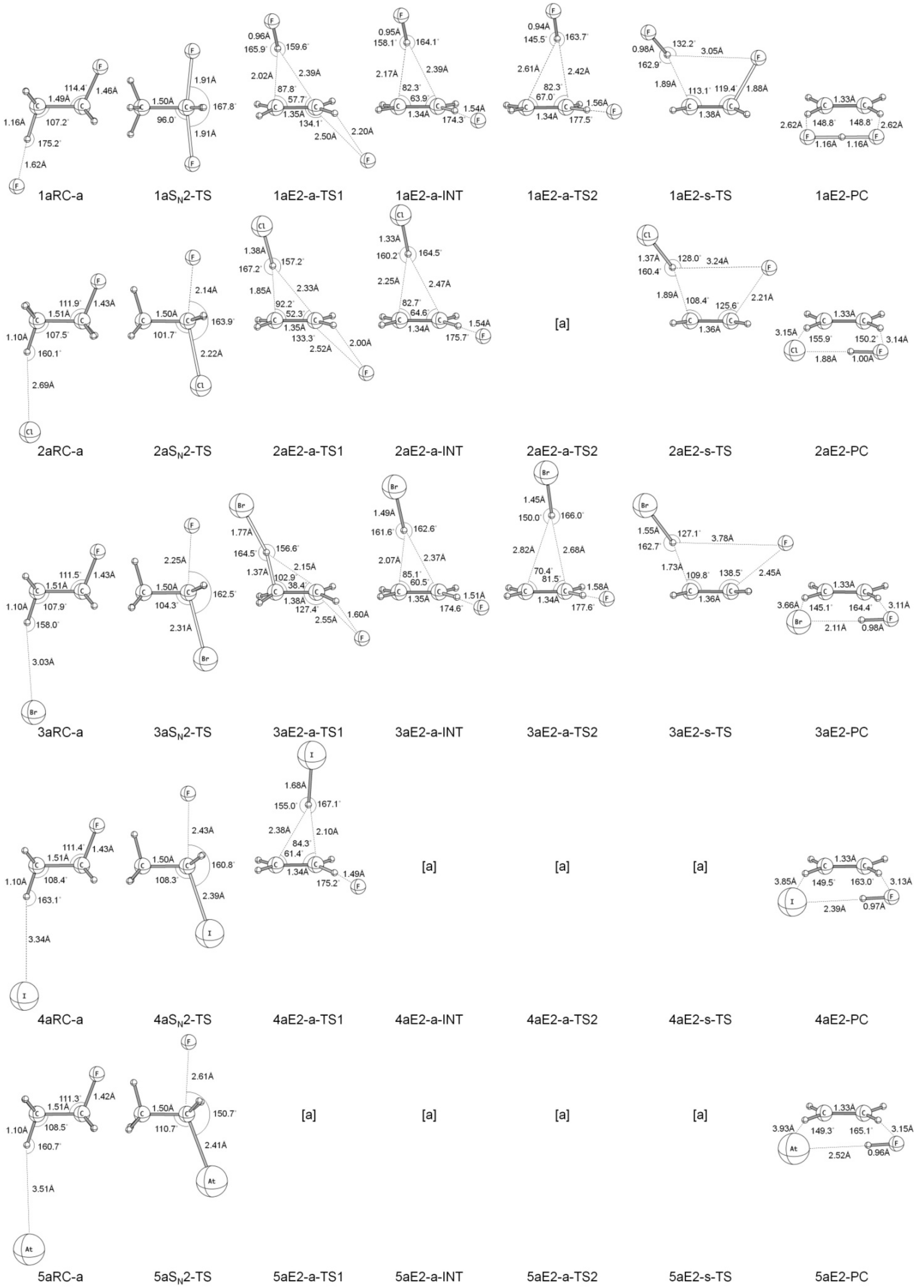
1aRC-a
1aS$_N$2-TS
1aE2-a-TS1
1aE2-a-INT
1aE2-a-TS2
1aE2-s-TS
1aE2-PC
[a]
2aRC-a
2aS$_N$2-TS
2aE2-a-TS1
2aE2-a-INT
2aE2-a-TS2
2aE2-s-TS
2aE2-PC
3aRC-a
3aS$_N$2-TS
3aE2-a-TS1
3aE2-a-INT
3aE2-a-TS2
3aE2-s-TS
3aE2-PC
[a]
[a]
[a]
4aRC-a
4aS$_N$2-TS
4aE2-a-TS1
4aE2-a-INT
4aE2-a-TS2
4aE2-s-TS
4aE2-PC
[a]
[a]
[a]
[a]
5aRC-a
5aS$_N$2-TS
5aE2-a-TS1
5aE2-a-INT
5aE2-a-TS2
5aE2-s-TS
5aE2-PC

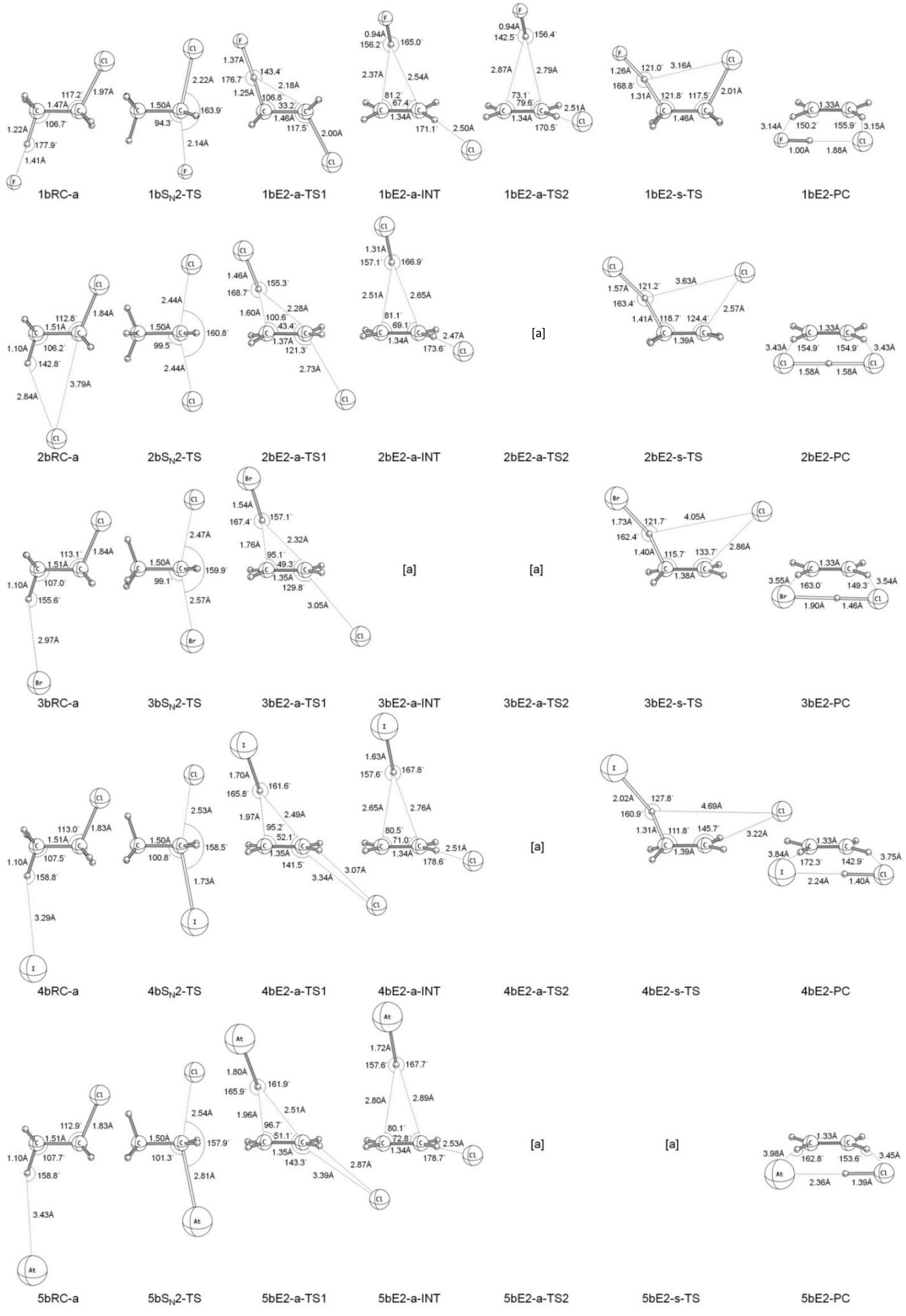
1bRC-a
1bS$_N$2-TS
1bE2-a-TS1
1bE2-a-INT
1bE2-a-TS2
1bE2-s-TS
1bE2-PC
[a]
2bRC-a
2bS$_N$2-TS
2bE2-a-TS1
2bE2-a-INT
2bE2-a-TS2
2bE2-s-TS
2bE2-PC
[a]
[a]
3bRC-a
3bS$_N$2-TS
3bE2-a-TS1
3bE2-a-INT
3bE2-a-TS2
3bE2-s-TS
3bE2-PC
[a]
4bRC-a
4bS$_N$2-TS
4bE2-a-TS1
4bE2-a-INT
4bE2-a-TS2
4bE2-s-TS
4bE2-PC
[a]
[a]
5bRC-a
5bS$_N$2-TS
5bE2-a-TS1
5bE2-a-INT
5bE2-a-TS2
5bE2-s-TS
5bE2-PC

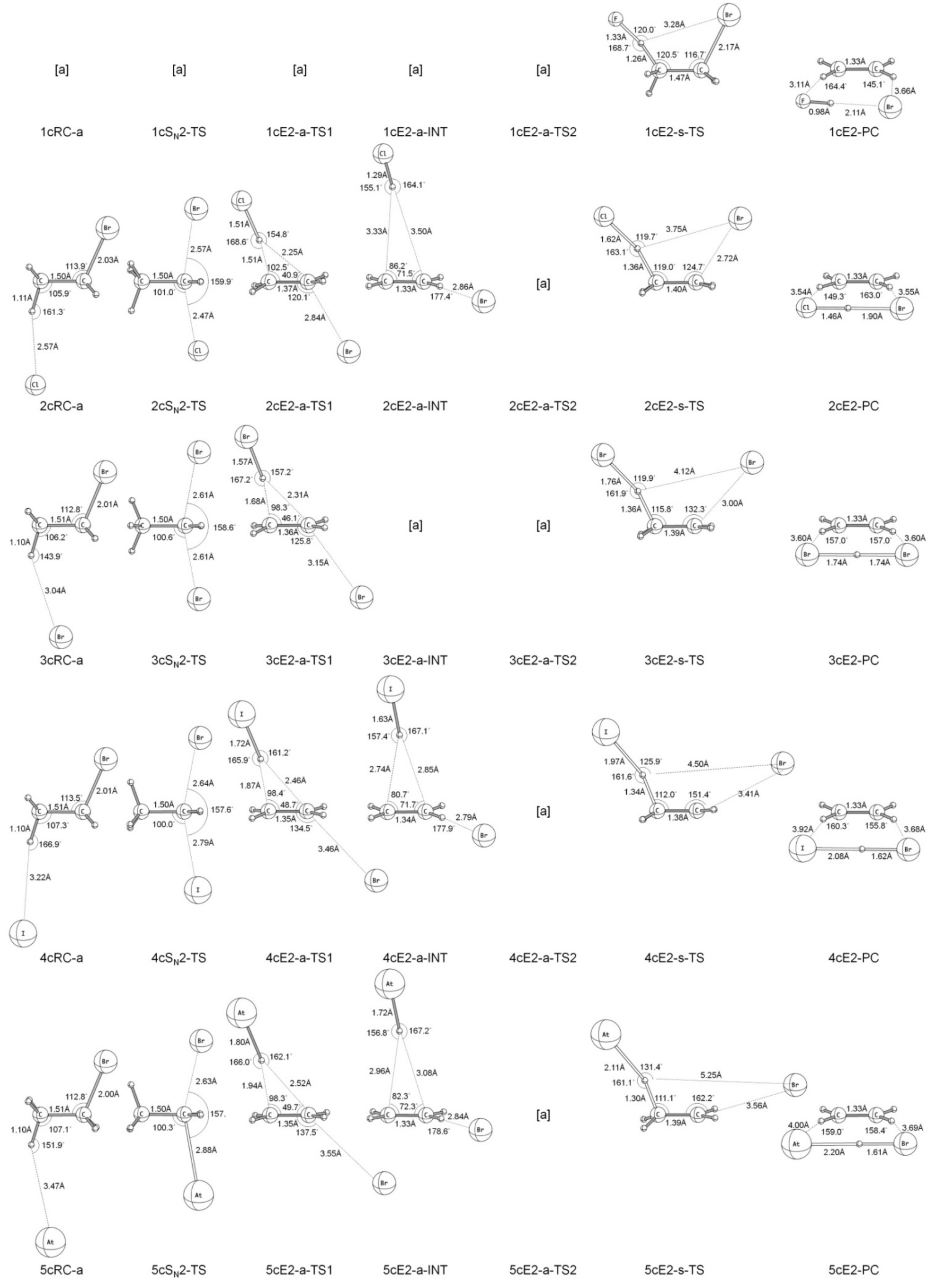
[a] [a] [a] [a] [a]
1cRC-a 1cS$_N$2-TS 1cE2-a-TS1 1cE2-a-INT 1cE2-a-TS2 1cE2-s-TS 1cE2-PC
[a]
2cRC-a 2cS$_N$2-TS 2cE2-a-TS1 2cE2-a-INT 2cE2-a-TS2 2cE2-s-TS 2cE2-PC
[a] [a]
3cRC-a 3cS$_N$2-TS 3cE2-a-TS1 3cE2-a-INT 3cE2-a-TS2 3cE2-s-TS 3cE2-PC
[a]
4cRC-a 4cS$_N$2-TS 4cE2-a-TS1 4cE2-a-INT 4cE2-a-TS2 4cE2-s-TS 4cE2-PC
[a]
5cRC-a 5cS$_N$2-TS 5cE2-a-TS1 5cE2-a-INT 5cE2-a-TS2 5cE2-s-TS 5cE2-PC

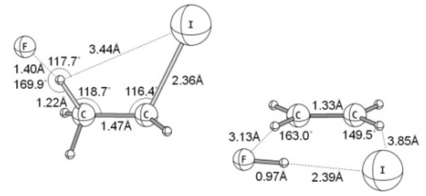

1dRC-a 1dS$_N$2-TS 1dE2-a-TS1 1dE2-a-INT 1dE2-a-TS2 1dE2-s-TS 1dE2-PC

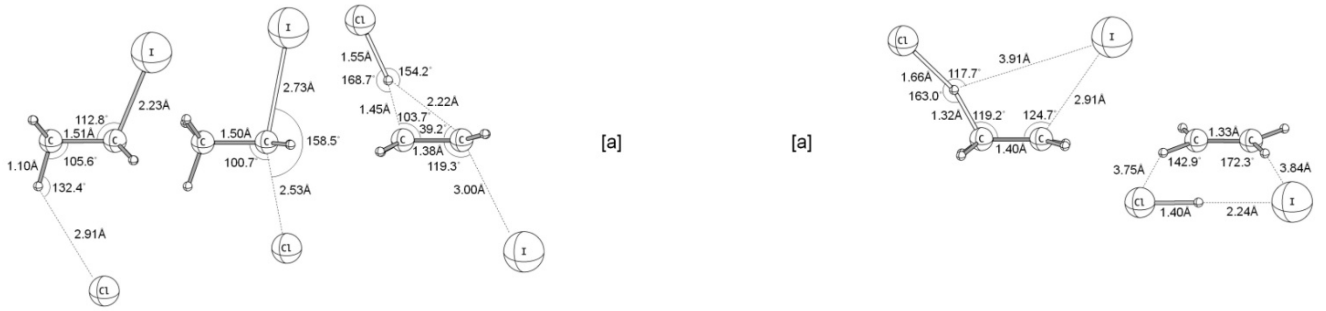

2dRC-a 2dS$_N$2-TS 2dE2-a-TS1 2dE2-a-INT 2dE2-a-TS2 2dE2-s-TS 2dE2-PC

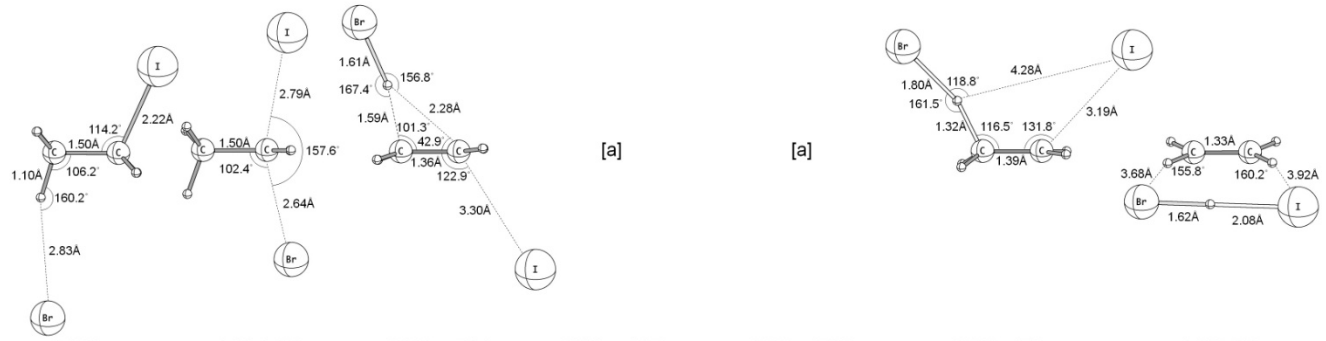

3dRC-a 3dS$_N$2-TS 3dE2-a-TS1 3dE2-a-INT 3dE2-a-TS2 3dE2-s-TS 3dE2-PC

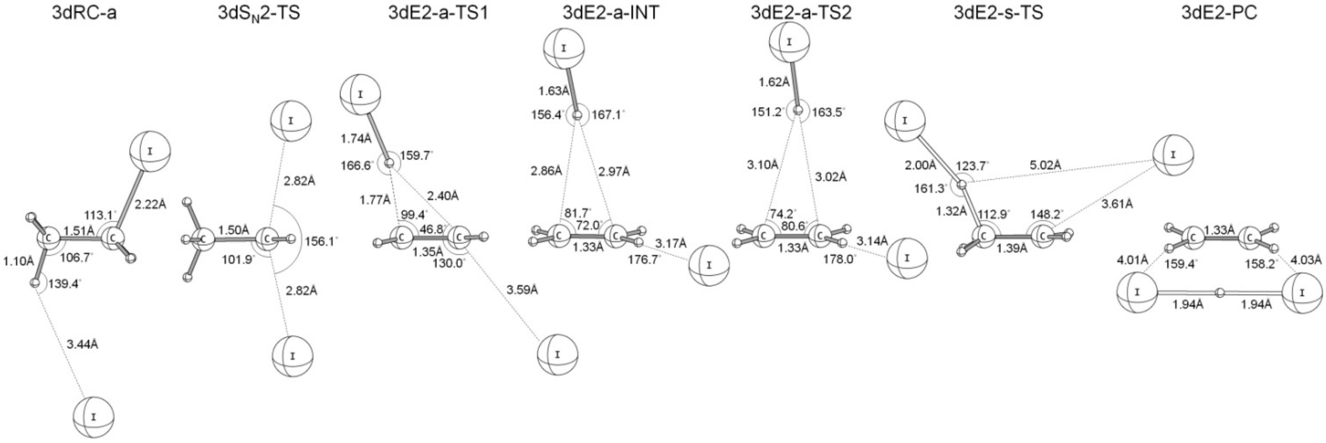

4dRC-a 4dS$_N$2-TS 4dE2-a-TS1 4dE2-a-INT 4dE2-a-TS2 4dE2-s-TS 4dE2-PC

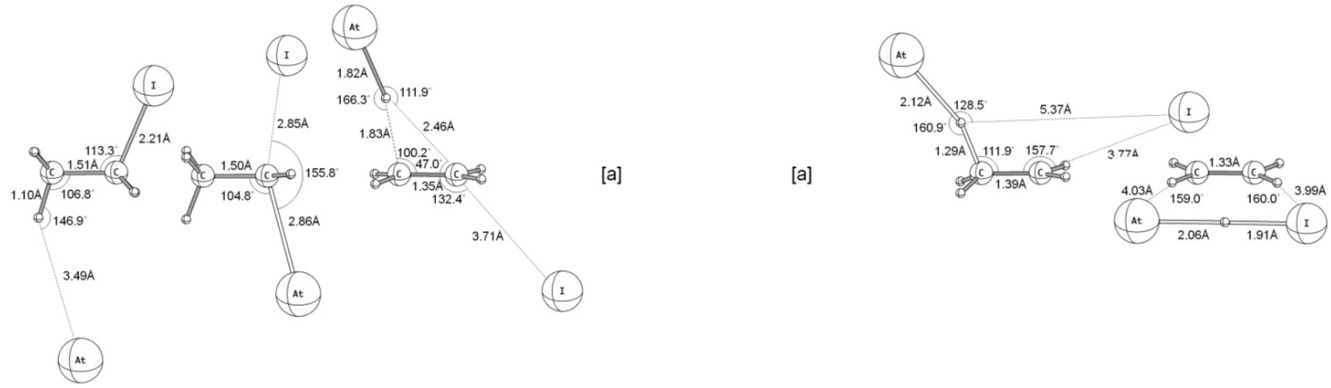

5dRC-a 5dS$_N$2-TS 5dE2-a-TS1 5dE2-a-INT 5dE2-a-TS2 5dE2-s-TS 5dE2-PC

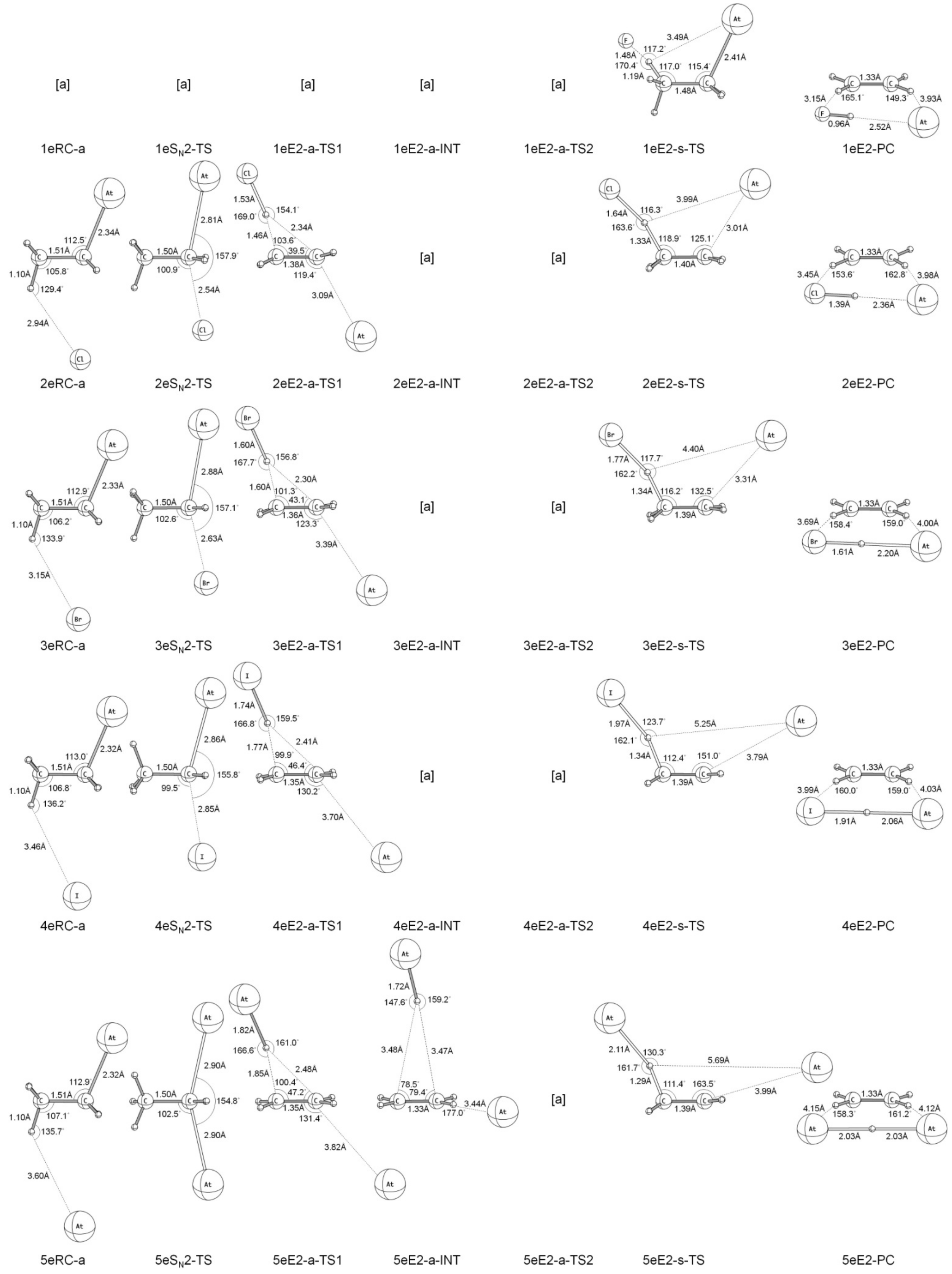

Figure S1. Structures (in Å, deg.) of stationary points in S_N2, *anti*-E2 and *syn*-E2 reactions of X^- + CH_3CH_2Y, computed at ZORA-OLYP/TZ2P. [a] no stationary point obtained.

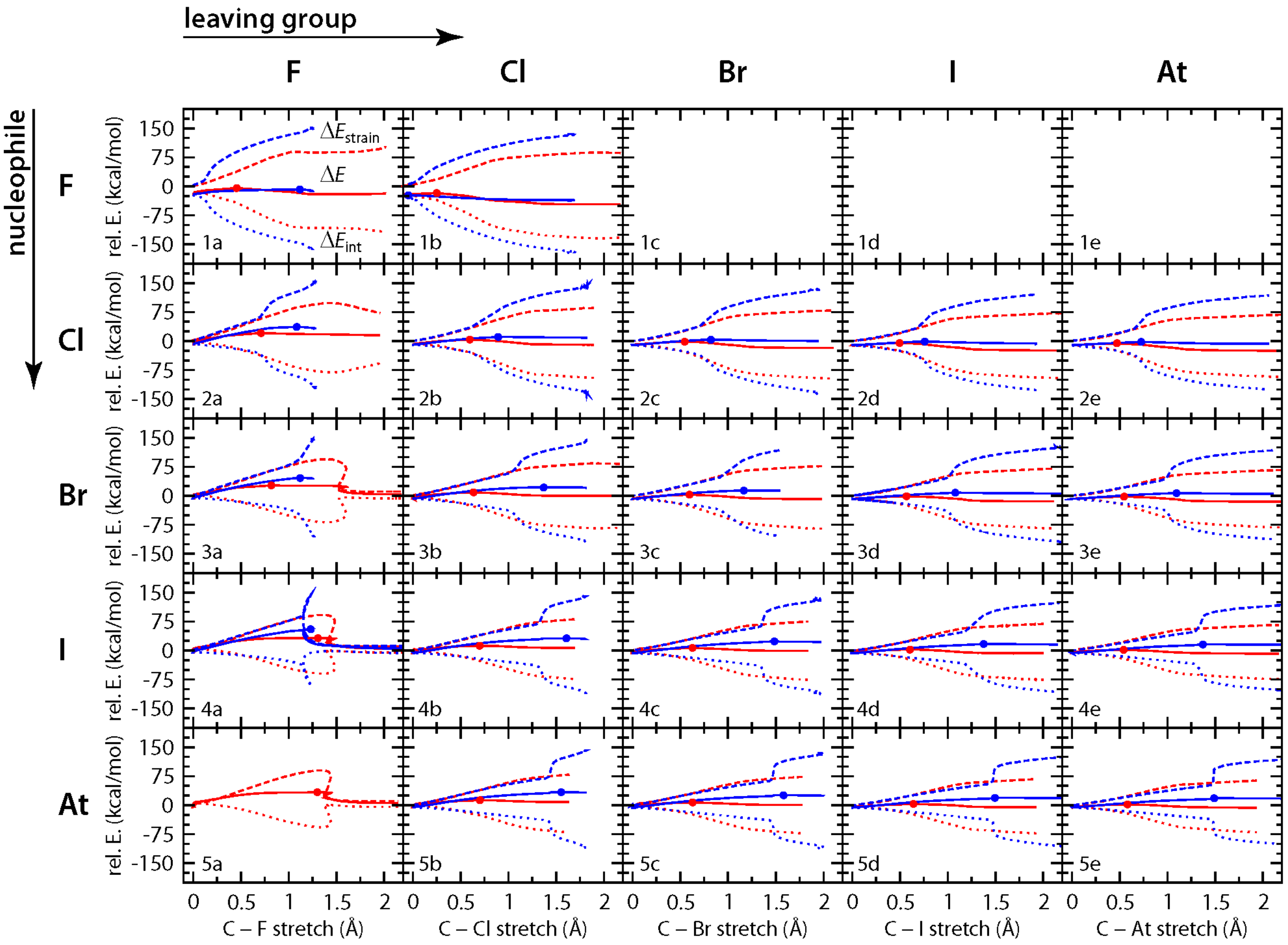

Figure S2. Activation-strain analysis of S_N2 (red) and *anti*-E2 (blue) reaction profiles (in kcal mol^{-1}) along the reaction coordinate projected onto the C–Y stretch (in Å), computed at ZORA-OLYP/TZ2P. Solid, dashed and dotted lines represent total, strain, and interaction energy, respectively. Dots indicate transition states.

Table S1. Cartesian coordinates (in Å) of all $[C_2H_5XY]^-$ intermediates and transition states, computed at ZORA-OLYP/TZ2P (RC-a = *anti* reactant complex; RC-s *syn* reactant complex; S_N2-TS = S_N2 transition state; E2-s-TS = *syn*-E2 transition state; E2-a-TS1 = first *anti*-E2 transition state; E2-a-INT = *anti*-E2 intermediate complex; E2-a-TS2 = second *anti*-E2 transition state; E2-PC = E2 product complex; S_N2-PC = S_N2 product complex). [a] Barrierless E2 reaction; [b] Nonexistent; [c] stationary point could not be obtained due to shallowness of PES.

1a RC-a	-1015.4 kcal/mol		NIMAG = 0
F	1.92511	-1.34537	0.00000
C	1.48143	0.04264	0.00000
C	0.00055	0.21691	0.00000
H	-0.20933	1.35619	0.00000
H	-0.45137	-0.23350	0.89397
H	-0.45137	-0.23350	-0.89397
H	1.94094	0.48544	0.89111
H	1.94094	0.48544	-0.89111
F	-0.36871	2.96468	0.00000

1a RC-s	-1010.6 kcal/mol		NIMAG = 0
F	2.55787	0.93337	0.22991
C	1.46177	0.27908	-0.40862
C	0.18352	0.41155	0.37984
H	-0.26777	1.44635	0.30134
H	0.34240	0.08502	1.41785
H	-0.55235	-0.27279	-0.07063
H	1.79779	-0.76287	-0.53848
H	1.35953	0.74975	-1.39510
F	-1.14074	2.86611	-0.19662

1a S_N2-TS	-999.5 kcal/mol		NIMAG = 1	-423.8 cm^{-1}
C	0.02203	-0.00006	0.00440	
C	1.52273	0.00000	0.00060	
F	-0.17811	1.89556	-0.00057	
F	-0.17810	-1.89493	-0.04875	
H	-0.54283	0.01154	-0.90831	
H	-0.50373	-0.01198	0.94242	
H	1.91565	0.01299	-1.02148	
H	1.89128	0.89050	0.51861	
H	1.89129	-0.90338	0.49582	

1a E2-s-TS	-1000.3 kcal/mol		NIMAG = 1	-159.4 cm^{-1}
F	2.55457	1.35292	-0.00048	
C	1.35534	-0.09578	0.00077	
C	-0.00645	0.14888	-0.00074	
H	-0.42934	1.99554	-0.00014	
H	-0.57731	0.13177	0.92648	
H	-0.57547	0.13050	-0.92908	
H	1.83020	-0.44204	0.91434	
H	1.83207	-0.44456	-0.91093	
F	-0.91845	2.84260	0.00033	

1a E2-a-TS1 -1003.3 kcal/mol NIMAG = 1 -83.2 cm^{-1}

F	-0.28274	-0.15924	0.26477
C	2.20327	-0.03350	0.01339
C	3.09817	0.97940	0.00047
H	4.55858	-0.41190	0.00856
H	3.41717	1.47057	0.91999
H	3.40940	1.45877	-0.92836
H	1.82429	-0.47969	-0.89876
H	1.79788	-0.46662	0.91922
F	5.39556	-0.88577	0.00608

1a E2-a-INT -1007.9 kcal/mol NIMAG = 0

C	0.14139	-0.25146	0.23875
C	1.47414	-0.14293	0.10307
H	-0.59058	-0.54393	-0.61245
H	-0.27166	-0.11003	1.24575
H	1.94837	-0.30057	-0.86853
H	2.15795	0.04923	0.93603
H	1.00258	1.97432	0.05684
F	1.14077	2.91127	0.14128
F	-1.57492	-1.06747	-1.67155

1a E2-a-TS2 -1007.6 kcal/mol NIMAG = 1 -72.1 cm^{-1}

C	-0.19042	-0.27081	-0.18724
C	1.14466	-0.17031	-0.26074
H	0.68376	2.11704	0.37850
H	-0.68805	-0.38655	0.77860
H	-0.85092	-0.25841	-1.05986
H	1.84447	-0.19091	0.65817
H	1.58871	-0.06756	-1.25892
F	0.49006	3.03822	0.36191
F	2.76241	-0.15751	1.91484

1a E2-PC -1036.8 kcal/mol NIMAG = 0

C	0.00000	-0.66687	-0.90721
C	0.00000	0.66687	-0.90721
H	0.00000	-1.23986	-1.83610
H	0.00000	-1.21202	0.03579
H	0.00000	1.23986	-1.83610
H	0.00000	1.21202	0.03579
H	0.00000	0.00000	2.65866
F	0.00000	-1.15702	2.65101
F	0.00000	1.15702	2.65101

1a S_N2-PC -1015.4 kcal/mol NIMAG = 0

F	1.92511	-1.34537	0.00000
C	1.48143	0.04264	0.00000
C	0.00055	0.21691	0.00000
H	-0.20933	1.35619	0.00000
H	-0.45137	-0.23350	0.89397
H	-0.45137	-0.23350	-0.89397
H	1.94094	0.48544	0.89111

H	1.94094	0.48544	-0.89111
F	-0.36871	2.96468	0.00000

2a RC-a	-1000.8 kcal/mol	NIMAG = 0	
C	0.111171	0.061689	0.051800
F	1.527942	-0.109721	-0.060187
C	-0.283321	1.513457	0.008678
H	-1.379952	1.562105	0.099856
H	0.018711	1.975835	-0.938332
H	0.170502	2.069833	0.837134
H	-0.334561	-0.501218	-0.774324
H	-0.183293	-0.407507	0.995816
CL	-3.932058	0.759901	0.360781

2a RC-s
[b]

2a S_N2-TS	-971.7 kcal/mol	NIMAG = 1	-336.1 cm^{-1}
C	0.163740	0.063150	0.014448
CL	2.382409	-0.008696	0.005665
C	-0.092949	1.539980	0.004630
F	-1.914036	-0.462737	0.033499
H	0.090247	-0.487115	0.934742
H	0.081448	-0.498320	-0.898515
H	0.319231	2.021179	0.897429
H	0.353800	2.014939	-0.874785
H	-1.178786	1.672859	-0.015790

2a E2-s-TS	-953.5 kcal/mol	NIMAG = 1	-157.3 cm^{-1}
F	2.775861	1.421456	-0.000131
C	1.231089	-0.152312	-0.000113
C	-0.115488	0.057396	0.000078
H	-0.430353	1.919515	0.000049
H	-0.688413	0.067528	0.926046
H	-0.688676	0.067542	-0.925727
H	1.779591	-0.330557	0.914818
H	1.779308	-0.330546	-0.915180
CL	-1.097097	3.111737	0.000321

2a E2-a-TS1	-956.1 kcal/mol	NIMAG = 1	-188.6 cm^{-1}
C	0.241403	0.272606	-0.225299
C	1.497908	0.665781	0.091780
H	1.034198	2.451182	0.014148
H	2.276624	0.771475	-0.664597
H	1.836366	0.695141	1.127034
H	-0.065290	0.184258	-1.263584
H	-0.530070	0.009031	0.491662
CL	0.986477	3.830090	0.010858
F	-1.019901	-1.881678	0.080510

2a E2-a-INT	-960.1 kcal/mol	NIMAG = 0	
C	0.158182	-0.350467	0.269513

C	1.489415	-0.238937	0.124760
H	-0.582601	-0.593060	-0.588900
H	-0.245196	-0.241638	1.284326
H	1.953768	-0.357515	-0.856964
H	2.181273	-0.074131	0.956559
H	1.013123	1.957803	0.075734
CL	1.174117	3.271532	0.152930
F	-1.576696	-1.019391	-1.680936

2a E2-a-TS2

[c]

2a E2-PC   -1006.4 kcal/mol   NIMAG = 0

C	0.244218	1.069686	-0.007695
C	1.576463	1.030172	0.006832
H	2.179904	1.938109	0.010403
H	2.103821	0.078788	0.015487
H	-0.343450	0.153096	-0.010796
H	-0.293412	2.018393	-0.016672
H	1.061895	-3.034911	-0.000910
F	2.059522	-3.065008	-0.003669
CL	-0.811908	-2.959228	0.004590

2a S_N2-PC   -977.5 kcal/mol   NIMAG = 0

C	-0.082391	0.012702	0.082165
CL	1.886461	-0.023091	-0.036845
C	-0.732207	1.326954	0.060276
H	-1.934447	1.124534	0.137699
H	-0.552665	1.872528	-0.873203
H	-0.448585	1.953848	0.913486
H	-0.332083	-0.619215	-0.770317
H	-0.222864	-0.535778	1.013730
F	-3.334234	0.942236	0.221872

3a RC-a   -955.4 kcal/mol   NIMAG = 0

C	0.031820	0.226486	-0.000117
C	1.537311	0.165351	0.000069
F	2.114928	1.471720	-0.000213
H	-0.341372	0.743021	0.891472
H	1.920185	-0.344210	0.889821
H	1.920418	-0.344709	-0.889297
H	-0.341162	0.742524	-0.892083
H	-0.348343	-0.803748	0.000152
BR	-0.256374	-3.833910	0.002739

3a RC-s

[b]

3a S_N2-TS   -962.3 kcal/mol   NIMAG = 1 -258.4 cm^{-1}

C	0.148480	0.096918	0.013553
BR	2.455813	-0.010394	0.001974
C	-0.153103	1.565300	0.005630

F	-2.024716	-0.479382	0.035510
H	0.023140	-0.456640	0.928572
H	0.013374	-0.466641	-0.894080
H	0.241498	2.064310	0.896763
H	0.263290	2.059334	-0.878360
H	-1.245272	1.645555	-0.006981

3a E2-s-TS -937.9 kcal/mol NIMAG = 1 -141.2 cm^{-1}

C	-0.167829	0.154956	0.002320
F	1.998747	-0.995279	-0.001592
C	-0.644435	1.429363	-0.001432
H	0.677640	2.551077	0.001335
H	-0.969082	1.908504	0.921676
H	-0.964263	1.904796	-0.928144
H	0.043215	-0.395079	0.910595
H	0.049030	-0.399812	-0.901632
BR	1.508114	3.858341	0.003233

3a E2-a-TS1 -942.7 kcal/mol NIMAG = 1 -312.0 cm^{-1}

C	-0.144739	-0.540281	0.037481
C	1.006838	-0.073310	-0.561429
F	2.533370	1.800982	0.260811
H	-0.241385	-0.436502	1.118866
H	1.895907	0.366597	-0.034710
H	1.022041	-0.013097	-1.652755
H	-1.092739	-0.531395	-0.507497
H	0.003343	-1.901733	-0.032350
BR	-0.256877	-3.651608	0.036332

3a E2-a-INT -944.4 kcal/mol NIMAG = 0

C	0.154392	-0.297038	0.266682
C	1.482223	-0.131221	0.119651
H	-0.585652	-0.545029	-0.597373
H	-0.244509	-0.231123	1.286767
H	1.945266	-0.209553	-0.866435
H	2.174044	0.016735	0.954242
H	1.046504	1.896619	0.106737
BR	1.201832	3.375961	0.174543
F	-1.556122	-0.999370	-1.667042

3a E2-a-TS2 -943.7 kcal/mol NIMAG = 1 -44.4 cm^{-1}

C	-0.149172	-0.423613	-0.178925
C	1.186476	-0.327740	-0.238778
H	0.634441	2.227497	0.366393
H	-0.656021	-0.554288	0.779980
H	-0.801287	-0.386532	-1.056979
H	1.879914	-0.363926	0.677894
H	1.640684	-0.203762	-1.229778
BR	0.352810	3.647022	0.341970
F	2.828873	-0.348601	1.938280

3a E2-PC -997.6 kcal/mol NIMAG = 0

C	0.343310	1.382830	-0.004966
C	1.648130	1.112451	0.002823
H	2.396566	1.904600	0.002027
H	2.007490	0.086080	0.009955
H	-0.393050	0.582308	-0.004252
H	-0.026311	2.408167	-0.012503
H	1.229400	-3.034279	-0.000069
F	2.210005	-3.016540	-0.002439
BR	-0.882093	-3.043766	0.004554

3a S_N2-PC

[a]

4a RC-a	-988.7 kcal/mol	NIMAG = 0	
C	0.479820	0.014523	-0.000231
F	1.905362	0.003275	-0.000002
C	-0.059390	1.422308	-0.000189
H	-1.155588	1.374150	-0.000365
H	0.267736	1.968661	-0.891628
H	0.267440	1.968518	0.891441
H	0.164589	-0.539297	-0.890597
H	0.164330	-0.539417	0.889967
I	-4.303862	0.265175	0.001049

4a RC-s

[b]

4a S_N2-TS	-951.5 kcal/mol	NIMAG = 1	-46.8 cm^{-1}
C	0.115400	0.144200	0.013500
I	2.504900	-0.016100	0.001800
C	-0.259800	1.598200	0.006200
F	-2.230300	-0.500400	0.036400
H	-0.098900	-0.409100	0.916200
H	-0.107600	-0.419900	-0.880400
H	0.111000	2.125500	0.891800
H	0.109400	2.116600	-0.885300
H	-1.356900	1.596900	0.007700

4a E2-s-TS

[c]

4a E2-a-TS1	-928.7 kcal/mol	NIMAG = 1	-83.5 cm^{-1}
F	0.914868	-0.972963	0.554153
C	2.622625	0.670566	-0.660426
C	3.184382	1.743455	-0.076245
H	4.388294	-0.305824	-0.086560
H	3.076886	1.911846	0.997682
H	3.768988	2.496394	-0.614402
H	2.764599	0.566975	-1.743464
H	1.913409	-0.084778	-0.111498
I	5.762749	-1.267237	0.044486

4a E2-a-INT

[c]

4a E2-a-TS2

[c]

4a E2-PC -988.1 kcal/mol NIMAG = 0

C	0.511578	1.527640	-0.010298
C	1.822826	1.291487	0.007183
H	2.550416	2.102561	0.014466
H	2.210628	0.275506	0.014803
H	-0.206396	0.710519	-0.017161
H	0.115779	2.542959	-0.018167
H	1.455846	-2.921652	-0.002356
F	2.421473	-2.847246	-0.005072
I	-0.927933	-3.075599	0.005106

4a S_N2-PC

[a]

5a RC-a -982.3 kcal/mol NIMAG = 0

C	0.700636	2.356187	0.010669
C	1.843346	1.375056	0.086743
H	2.513012	1.484334	-0.773265
H	1.430050	0.358966	0.082126
H	2.421034	1.514284	1.007008
H	0.117935	2.227894	-0.907265
H	0.029289	2.262829	0.870497
F	1.172405	3.700426	0.007796
AT	-0.889227	-2.268795	-0.010456

5a RC-s

[b]

5a S_N2-TS -943.7 kcal/mol NIMAG = 1 -86.6 cm^{-1}

C	-0.392500	0.177000	-0.229600
F	2.175300	-0.150100	0.078100
C	-0.287600	1.661200	-0.005300
AT	-2.660300	-0.574500	0.093200
H	0.220700	-0.423700	0.440500
H	-0.241500	-0.130600	-1.261000
H	-0.569900	1.943800	1.014300
H	0.777500	1.891200	-0.139000
H	-0.891600	2.245600	-0.708900

5a E2-s-TS

[c]

5a E2-a-TS1

[c]

5a E2-a-INT			
[c]			

5a E2-a-TS2			
[c]			

5a E2-PC	-980.5 kcal/mol	NIMAG = 0	
C	0.581054	1.615856	-0.008319
C	1.890062	1.367627	0.004653
H	2.625079	2.171847	0.009534
H	2.269181	0.348393	0.010736
H	-0.144761	0.805590	-0.012920
H	0.194445	2.634631	-0.014581
H	1.607957	-2.868125	-0.002815
F	2.569136	-2.784711	-0.004541
AT	-0.906331	-3.048292	0.004919

5a S_N2*-PC*			
[a]			

1b RC-a	-977.5 kcal/mol	NIMAG = 0	
C	-0.082391	0.012702	0.082165
CL	1.886461	-0.023091	-0.036845
C	-0.732207	1.326954	0.060276
H	-1.934447	1.124534	0.137699
H	-0.552665	1.872528	-0.873203
H	-0.448585	1.953848	0.913486
H	-0.332083	-0.619215	-0.770317
H	-0.222864	-0.535778	1.013730
F	-3.334234	0.942236	0.221872

1b RC-s	-970.7 kcal/mol	NIMAG = 0	
C	0.189152	0.463199	0.366179
C	1.460476	0.221471	-0.414139
CL	2.917643	1.133842	0.243019
H	-0.285092	1.457173	0.105425
H	1.803488	-0.818594	-0.414720
H	1.358723	0.575847	-1.442993
H	-0.531988	-0.302484	0.037537
H	0.353464	0.321340	1.442212
F	-1.175163	2.698359	-0.696584

1b S_N2*-TS* -	971.7 kcal/mol	NIMAG = 1	-336.1 cm^{-1}
C	0.163740	0.063150	0.014448
CL	2.382409	-0.008696	0.005665
C	-0.092949	1.539980	0.004630
F	-1.914036	-0.462737	0.033499
H	0.090247	-0.487115	0.934742
H	0.081448	-0.498320	-0.898515
H	0.319231	2.021179	0.897429
H	0.353800	2.014939	-0.874785
H	-1.178786	1.672859	-0.015790

1b E2-s-TS	-966.8 kcal/mol	NIMAG = 1	-423.4 cm^{-1}
CL	-0.085036	-0.095483	0.012502
C	1.915105	0.092135	-0.046175
C	2.469734	1.444156	0.042992
H	1.702997	2.509809	0.003284
H	3.120011	1.660249	-0.813245
H	3.010799	1.602043	0.983266
H	2.068749	-0.423773	-0.993192
H	2.119317	-0.579645	0.786897
F	1.182050	3.651270	-0.025582

1b E2-a-TS1	-977.5 kcal/mol	NIMAG = 1	-142.7 cm^{-1}
C	-0.316365	0.188253	-0.019157
C	1.060746	0.665920	0.037468
H	1.011709	1.910724	0.015696
H	1.658382	0.358486	-0.828029
H	1.574908	0.386695	0.963961
H	-0.839629	0.426559	-0.944861
H	-0.923119	0.454898	0.845848
F	1.037649	3.276117	-0.004342
CL	-0.609285	-1.793609	-0.001425

1b E2-a-INT	-991.2 kcal/mol	NIMAG = 0	
C	0.334682	-0.081675	0.416993
C	1.656008	-0.004219	0.223211
H	-0.368227	-0.355953	-0.380732
H	-0.084965	0.091943	1.409338
H	2.087198	-0.203812	-0.757110
H	2.356311	0.217530	1.029060
H	1.142677	2.308780	0.160604
F	1.304929	3.229981	0.265359
CL	-1.966579	-1.330896	-2.030219

1b E2-a-TS2	-990.9 kcal/mol	NIMAG = 1	-47.7 cm^{-1}
H	-0.029683	-0.018791	0.396368
CL	2.443614	0.021052	-0.001392
C	-1.776323	1.124967	-0.022024
C	-1.124812	0.040778	0.406508
H	-1.198187	-0.469466	-2.339077
H	-1.228054	1.993496	-0.385155
H	-2.864191	1.196993	-0.019691
H	-1.678399	-0.823121	0.778727
F	-1.566260	-0.766157	-3.145775

1b E2-PC	-1006.4 kcal/mol	NIMAG = 0	
C	0.244218	1.069686	-0.007695
C	1.576463	1.030172	0.006832
H	2.179904	1.938109	0.010403
H	2.103821	0.078788	0.015487
H	-0.343450	0.153096	-0.010796
H	-0.293412	2.018393	-0.016672

H	1.061895	-3.034911	-0.000910	
F	2.059522	-3.065008	-0.003669	
CL	-0.811908	-2.959228	0.004590	

1b S_N2-PC	-1000.8 kcal/mol		NIMAG = 0	
C	0.111171	0.061689	0.051800	
F	1.527942	-0.109721	-0.060187	
C	-0.283321	1.513457	0.008678	
H	-1.379952	1.562105	0.099856	
H	0.018711	1.975835	-0.938332	
H	0.170502	2.069833	0.837134	
H	-0.334561	-0.501218	-0.774324	
H	-0.183293	-0.407507	0.995816	
CL	-3.932058	0.759901	0.360781	

2b RC-a	-961.0 kcal/mol		NIMAG = 0	
CL	-0.16676	-0.16062	0.00000	
C	1.62856	0.25738	0.00000	
C	1.88280	1.74509	0.00000	
H	2.97384	1.86962	0.00000	
H	1.46224	2.22476	0.89009	
H	1.46224	2.22476	-0.89009	
H	2.03985	-0.22798	0.88411	
H	2.03985	-0.22798	-0.88411	
CL	5.41722	0.42020	0.00000	

2b RC-s
[b]

2b S_N2-TS	-947.3 kcal/mol		NIMAG = 1	-326.4 cm^{-1}
C	0.15292	-0.00020	0.01550	
C	1.65223	-0.00005	0.00435	
CL	-0.25157	2.40326	-0.00025	
CL	-0.25156	-2.40248	-0.06132	
H	-0.42430	0.01129	-0.88879	
H	-0.37891	-0.01207	0.95014	
H	2.04197	0.01293	-1.01685	
H	2.02689	0.88316	0.52882	
H	2.02689	-0.89631	0.50621	

2b E2-s-TS	-931.7 kcal/mol		NIMAG = 1	-616.3 cm^{-1}
C	0.32462	-0.02706	0.00017	
C	1.67141	0.32709	-0.00045	
CL	-1.61795	1.65270	0.00031	
CL	2.81166	3.04929	-0.00324	
H	2.01194	1.69436	-0.00145	
H	2.22780	0.14713	-0.92111	
H	2.22849	0.14781	0.91997	
H	-0.18883	-0.29369	-0.91388	
H	-0.18814	-0.29325	0.91473	

2b E2-a-TS1	-940.6 kcal/mol		NIMAG = 1	-159.6 cm^{-1}

C	-0.13942	0.36316	-0.01533
C	1.16596	0.76777	0.03919
H	0.98345	2.35267	0.00684
H	1.78827	0.71416	-0.85266
H	1.70229	0.74382	0.98647
H	-0.66738	0.28625	-0.95772
H	-0.75326	0.31643	0.87549
CL	1.10502	3.81133	-0.00814
CL	-0.80418	-2.28884	-0.00173

2b E2-a-INT	-943.8 kcal/mol	NIMAG = 0	
C	0.02818	-0.17228	-0.05777
C	1.29399	-0.52151	0.19124
H	1.63343	1.93753	-0.17383
H	1.92193	-0.92830	-0.60039
H	1.74426	-0.43989	1.18087
H	-0.42634	-0.28518	-1.04987
H	-0.60084	0.22083	0.74353
CL	2.21448	3.10153	-0.06117
CL	-1.39132	-0.80607	-3.25987

2b E2-a-TS2	-943.8 kcal/mol	NIMAG = 1	-163.4 cm^{-1}
C	-0.08631	-0.17514	-0.04218
C	1.19377	-0.55413	-0.04673
H	1.39242	2.53018	-0.20576
H	-0.59082	0.07763	0.88994
H	-0.68212	-0.10714	-0.95290
H	1.79703	-0.62202	0.86680
H	1.68672	-0.81082	-0.98613
CL	1.40309	3.73749	-0.67656
CL	3.26219	-0.64710	2.89561

2b E2-PC	-963.1 kcal/mol	NIMAG = 0	
C	-0.00001	-0.07717	0.00000
C	1.33263	-0.07717	0.00000
H	0.66629	4.26419	0.00000
H	-0.56032	0.85493	0.00000
H	-0.57072	-1.00578	0.00000
H	1.89293	0.85494	0.00000
H	1.90335	-1.00577	0.00000
CL	-0.91474	4.26262	0.00000
CL	2.24733	4.26263	0.00000

2b S_N2-PC	-961.0 kcal/mol	NIMAG = 0
CL	-0.16676	-0.16062	0.00000
C	1.62856	0.25738	0.00000
C	1.88280	1.74509	0.00000
H	2.97384	1.86962	0.00000
H	1.46224	2.22476	0.89009
H	1.46224	2.22476	-0.89009
H	2.03985	-0.22798	0.88411
H	2.03985	-0.22798	-0.88411

CL	5.41722	0.42020	0.00000

3b RC-a -955.3 kcal/mol NIMAG = 0

C	0.169297	0.190368	0.073220
CL	1.969796	-0.156527	-0.051773
C	-0.158323	1.661422	-0.005755
H	-1.250398	1.751882	0.078783
H	0.163265	2.092507	-0.959227
H	0.310816	2.220377	0.810232
H	-0.287485	-0.374307	-0.739560
H	-0.140665	-0.247008	1.022289
BR	-4.027631	0.756360	0.382908

3b RC-s

[b]

3b S_N2-TS -939.1 kcal/mol NIMAG = 1 -296.9 cm^{-1}

C	0.226985	0.190263	0.004171
CL	2.694278	0.002750	-0.013038
C	0.052009	1.679005	-0.008667
BR	-2.247260	-0.508747	0.071826
H	0.318934	-0.356421	0.924391
H	0.288115	-0.363856	-0.915311
H	-0.362358	2.034134	0.938573
H	1.022069	2.158956	-0.167666
H	-0.623977	1.980486	-0.813418

3b E2-s-TS -919.8 kcal/mol NIMAG = 1 -494.8 cm^{-1}

CL	-0.476677	-0.701013	0.000828
C	2.182231	0.361295	0.000608
C	2.698520	1.645047	-0.001839
H	1.756372	2.676183	-0.003766
H	3.162362	1.998264	-0.923428
H	3.162257	2.001796	0.918424
H	1.988427	-0.189005	-0.911335
H	1.988016	-0.185288	0.914660
BR	1.029133	4.248373	-0.007236

3b E2-a-TS1 -926.2 kcal/mol NIMAG = 1 -56.0 cm^{-1}

C	0.161814	0.316685	0.004798
C	1.452931	0.719797	0.056044
H	1.081220	2.443672	0.012468
H	2.067125	0.756371	-0.841627
H	1.985362	0.783883	1.003067
H	-0.357882	0.161024	-0.932994
H	-0.438822	0.188075	0.897274
BR	1.093790	3.988246	-0.012001
CL	-1.005268	-2.504476	-0.009541

3b E2-a-INT

[c]

3b E2-a-TS2

[c]

3b E2-PC	-952.3 kcal/mol		NIMAG = 0
C	-0.719214	1.810196	-0.008110
C	0.608778	1.700744	0.009166
H	1.251330	2.580955	0.018441
H	1.095446	0.728249	0.014528
H	-1.353920	0.926923	-0.017074
H	-1.214024	2.781152	-0.013815
H	-0.208373	-2.678942	0.001810
BR	1.685432	-2.776461	-0.004583
CL	-1.665228	-2.602821	0.005339

3b S_N2-PC	-947.7 kcal/mol		NIMAG = 0
C	-0.065308	0.097319	0.000012
C	1.437379	0.064525	0.000532
BR	2.299658	1.897614	-0.000129
H	-0.463538	0.592590	0.891321
H	1.849588	-0.409267	0.891096
H	1.850217	-0.410191	-0.889242
H	-0.462949	0.592072	-0.891845
H	-0.392341	-0.959643	0.000238
CL	-0.325173	-3.529660	0.001951

4b RC-a	-948.4 kcal/mol		NIMAG = 0
C	0.409161	0.149722	0.057444
CL	2.209345	-0.173372	-0.071570
C	0.065085	1.617468	-0.033222
H	-1.025417	1.707343	0.053818
H	0.378795	2.043115	-0.991372
H	0.531663	2.188091	0.775676
H	-0.043948	-0.427310	-0.749099
H	0.108142	-0.282839	1.011933
I	-4.161471	0.763362	0.407329

4b RC-s

[b]

4b S_N2-TS	-929.6 kcal/mol		NIMAG = 1	-276.2 cm^{-1}
C	0.046900	0.127200	-0.008300	
CL	2.580100	0.053400	-0.017800	
C	-0.188800	1.607200	0.006200	
I	-2.520600	-0.800400	0.012300	
H	0.196000	-0.422600	0.903400	
H	0.180400	-0.402300	-0.934800	
H	-0.693300	1.919200	0.924500	
H	0.776200	2.120300	-0.052400	
H	-0.800100	1.917900	-0.845400	

4b E2-s-TS	-907.0 kcal/mol		NIMAG = 1	-113.4 cm^{-1}
CL	-1.916745	-2.919611	-0.038212	

C	0.741662	-1.113183	0.198216
C	1.341364	0.095011	-0.134853
H	0.507105	1.094534	-0.037566
H	1.617136	0.243630	-1.180375
H	2.052105	0.507934	0.583091
H	0.234126	-1.770932	-0.504662
H	0.661870	-1.428495	1.235481
I	-0.220422	2.983032	-0.009035

4b E2-a-TS1 -910.8 kcal/mol NIMAG = 1 -31.3 cm^{-1}

C	0.459700	0.133300	-0.010200
C	1.711100	0.623500	0.063500
H	1.166000	2.517100	0.009500
H	2.327500	0.749100	-0.824900
H	2.210900	0.768000	1.019500
H	-0.024800	-0.092900	-0.954200
H	-0.141500	-0.078100	0.867700
I	1.111200	4.214200	-0.012400
CL	-1.219700	-2.752300	-0.006800

4b E2-a-INT -912.4 kcal/mol NIMAG = 0

C	0.343516	-0.586769	0.502573
C	1.656199	-0.501092	0.268171
H	-0.387714	-0.772252	-0.293987
H	-0.040270	-0.478442	1.518427
H	2.050054	-0.616494	-0.740821
H	2.384919	-0.328375	1.060520
H	1.020303	2.065779	0.124205
I	1.242535	3.683808	0.195092
CL	-2.024137	-1.244956	-2.132326

4b E2-a-TS2

[c]

4b E2-PC -941.6 kcal/mol NIMAG = 0

C	0.099671	1.564038	-0.006114
C	1.426826	1.446162	0.005890
H	2.073936	2.322600	0.008028
H	1.913076	0.473573	0.013573
H	-0.542474	0.686307	-0.008379
H	-0.390112	2.537194	-0.014382
H	0.917506	-3.103809	0.000284
I	3.157329	-3.162537	-0.006929
CL	-0.481345	-3.062990	0.003461

4b S_N2-PC -935.2 kcal/mol NIMAG = 0

C	-0.477406	-0.011449	0.007668
CL	3.051918	0.094961	-0.023685
C	-0.411342	1.494885	0.006641
I	-2.573308	-0.784510	0.012120
H	-0.037707	-0.463514	0.893310
H	-0.040293	-0.464431	-0.878746

H	-0.877218	1.929063	0.897227
H	0.658477	1.743250	0.002883
H	-0.882386	1.927822	-0.881821

5b RC-a	-942.0 kcal/mol		NIMAG = 0
C	0.677947	0.068952	-0.015623
CL	2.507884	0.028068	-0.097218
C	0.116984	1.470350	-0.075009
H	-0.976067	1.395097	-0.008554
H	0.381798	1.968155	-1.012954
H	0.475137	2.079220	0.760604
H	0.340978	-0.543330	-0.852822
H	0.419605	-0.435220	0.915957
AT	-4.055141	-0.018132	0.510387

5b RC-s
[b]

5b S_N2-TS	-923.2 kcal/mol		NIMAG = 1	-265.8 cm^{-1}
C	0.107034	-0.219922	-0.010999	
C	1.604995	-0.173811	0.008012	
AT	-0.526750	2.521403	0.003016	
CL	-0.294671	-2.724046	-0.017006	
H	-0.460016	-0.294741	0.899433	
H	-0.432524	-0.285331	-0.939182	
H	1.995546	0.388762	-0.844355	
H	1.977140	0.289386	0.925784	
H	1.992434	-1.196379	-0.045551	

5b E2-s-TS
[c]

5b E2-a-TS1	-902.3 kcal/mol		NIMAG = 1	-44.8 cm^{-1}
C	-0.120319	1.147633	0.154893	
C	1.008255	0.454788	-0.087425	
CL	-3.496606	0.987485	-0.028019	
H	2.237406	1.984588	-0.012573	
H	1.321709	0.217741	-1.102188	
H	1.563915	-0.030800	0.712744	
H	-0.749287	1.560144	-0.627385	
H	-0.480552	1.324789	1.163694	
AT	3.667061	3.070248	-0.007413	

5b E2-a-INT	-903.8 kcal/mol		NIMAG = 0
C	0.240201	0.030897	0.806362
C	1.047710	-0.568913	-0.072473
CL	-2.873722	1.609659	-0.184689
H	2.264318	1.947671	0.057107
H	0.800308	-0.588885	-1.133035
H	1.971609	-1.066664	0.222913
H	-0.693391	0.523427	0.510291
H	0.494110	0.039912	1.867646

AT	3.535954	3.109116	-0.026080

5b E2-a-TS2

[c]

5b E2-PC -934.2 kcal/mol NIMAG = 0

C	0.722356	1.619723	-0.013832
C	2.051556	1.530558	0.010143
H	2.684416	2.417404	0.015978
H	2.550154	0.564136	0.024456
H	0.094760	0.731401	-0.019365
H	0.216941	2.584779	-0.028585
H	1.215532	-2.948149	0.002295
CL	2.607460	-2.881010	-0.006315
AT	-1.140854	-3.057081	0.005751

5b S_N2-PC -927.7 kcal/mol NIMAG = 0

C	0.058156	0.279508	-0.002961
C	1.548725	0.061416	0.008437
AT	-0.515649	2.550644	0.003086
CL	-0.319263	-3.175966	-0.020889
H	-0.450182	-0.101576	0.878816
H	-0.435334	-0.092712	-0.896827
H	2.036906	0.486704	-0.874362
H	2.021684	0.476744	0.904158
H	1.691644	-1.027426	0.003652

1c RC-a

[a]

1c RC-s

[a]

1c S_N2-TS

[a]

1c E2-s-TS -956.3 kcal/mol NIMAG = 1 -272.6 cm^{-1}

C	0.437651	-0.054732	-0.185270
BR	2.552680	-0.479129	0.018433
C	-0.003517	1.294285	0.184846
H	0.768708	2.278337	0.046718
H	-0.364008	1.342008	1.219173
H	-0.798775	1.613391	-0.503768
H	0.120357	-0.890597	0.437341
H	0.338734	-0.302453	-1.241847
F	1.368322	3.461905	-0.072581

1c E2-a-TS1

[a]

1c E2-a-INT

[a]

1c E2-a-TS2

[a]

1c E2-PC -997.6 kcal/mol NIMAG = 0

C	0.343310	1.382830	-0.004966
C	1.648130	1.112451	0.002823
H	2.396566	1.904600	0.002027
H	2.007490	0.086080	0.009955
H	-0.393050	0.582308	-0.004252
H	-0.026311	2.408167	-0.012503
H	1.229400	-3.034279	-0.000069
F	2.210005	-3.016540	-0.002439
BR	-0.882093	-3.043766	0.004554

1c S_N2-PC -995.4 kcal/mol NIMAG = 0

C	0.031820	0.226486	-0.000117
C	1.537311	0.165351	0.000069
F	2.114928	1.471720	-0.000213
H	-0.341372	0.743021	0.891472
H	1.920185	-0.344210	0.889821
H	1.920418	-0.344709	-0.889297
H	-0.341162	0.742524	-0.892083
H	-0.348343	-0.803748	0.000152
BR	-0.256374	-3.833910	0.002739

2c RC-a -947.7 kcal/mol NIMAG = 0

C	-0.065308	0.097319	0.000012
C	1.437379	0.064525	0.000532
BR	2.299658	1.897614	-0.000129
H	-0.463538	0.592590	0.891321
H	1.849588	-0.409267	0.891096
H	1.850217	-0.410191	-0.889242
H	-0.462949	0.592072	-0.891845
H	-0.392341	-0.959643	0.000238
CL	-0.325173	-3.529660	0.001951

2c RC-s

[b]

2c S_N2-TS -939.1 kcal/mol NIMAG = 1 -296.9 cm^{-1}

C	0.226985	0.190263	0.004171
CL	2.694278	0.002750	-0.013038
C	0.052009	1.679005	-0.008667
BR	-2.247260	-0.508747	0.071826
H	0.318934	-0.356421	0.924391
H	0.288115	-0.363856	-0.915311
H	-0.362358	2.034134	0.938573
H	1.022069	2.158956	-0.167666
H	-0.623977	1.980486	-0.813418

2c E2-s-TS	-924.4 kcal/mol	NIMAG = 1	-548.0 cm^{-1}
C	0.092258	0.218291	-0.000301
BR	2.687492	-0.588536	-0.000411
C	-0.325633	1.552250	0.000811
H	0.612354	2.536735	0.001844
H	-0.794215	1.908311	0.919793
H	-0.793727	1.909965	-0.917783
H	0.137875	-0.358994	0.914005
H	0.138478	-0.357268	-0.915615
CL	1.339218	3.981612	0.003178

2c E2-a-TS1	-933.9 kcal/mol	NIMAG = 1	-254.0 cm^{-1}
C	0.132490	0.100262	0.004976
C	1.499252	-0.044746	-0.003202
H	1.979738	1.385429	-0.000833
H	1.997192	-0.339802	-0.925750
H	2.007615	-0.345051	0.911933
H	-0.416033	0.291828	-0.909614
H	-0.405554	0.287455	0.926640
BR	-1.543412	-2.194870	0.011073
CL	2.733209	2.692642	0.000220

2c E2-a-INT	-937.6 kcal/mol	NIMAG = 0	
C	0.392571	-0.367084	0.496337
C	1.710844	-0.324815	0.292860
H	-0.313403	-0.658295	-0.284833
H	-0.026122	-0.121252	1.473052
H	2.135715	-0.582624	-0.676142
H	2.415128	-0.052257	1.079105
H	1.378723	2.987974	0.278482
CL	1.724399	4.203049	0.555618
BR	-2.192741	-1.525635	-2.257173

2c E2-a-TS2
[c]

2c E2-PC	-952.3 kcal/mol	NIMAG = 0	
C	-0.719214	1.810196	-0.008110
C	0.608778	1.700744	0.009166
H	1.251330	2.580955	0.018441
H	1.095446	0.728249	0.014528
H	-1.353920	0.926923	-0.017074
H	-1.214024	2.781152	-0.013815
H	-0.208373	-2.678942	0.001810
BR	1.685432	-2.776461	-0.004583
CL	-1.665228	-2.602821	0.005339

2c S_N2-PC	-955.3 kcal/mol	NIMAG = 0	
C	0.169297	0.190368	0.073220
CL	1.969796	-0.156527	-0.051773
C	-0.158323	1.661422	-0.005755
H	-1.250398	1.751882	0.078783

H	0.163265	2.092507	-0.959227
H	0.310816	2.220377	0.810232
H	-0.287485	-0.374307	-0.739560
H	-0.140665	-0.247008	1.022289
BR	-4.027631	0.756360	0.382908

3c RC-a	-942.1 kcal/mol	NIMAG = 0	
C	0.026712	0.013995	-0.874515
C	1.473959	0.009710	-0.452315
BR	-0.321923	0.036455	3.102441
BR	-0.201756	0.036704	-2.874372
H	-0.510631	0.897763	-0.534288
H	-0.510198	-0.878152	-0.555897
H	2.002080	0.902750	-0.801193
H	1.472812	0.002062	0.646230
H	2.001558	-0.877900	-0.815654

3c RC-s
[b]

3c S_N2-TS	-931.0 kcal/mol	NIMAG = 1	-267.2 cm^{-1}
C	0.184571	-0.013766	-0.000001
C	1.683550	-0.006077	0.000004
BR	-0.296166	0.040737	2.562314
BR	-0.296167	0.040738	-2.562315
H	-0.391329	0.892903	-0.000006
H	-0.352341	-0.947013	0.000002
H	2.077289	1.013320	-0.000031
H	2.061423	-0.522629	0.886897
H	2.061414	-0.522677	-0.886867

3c E2-s-TS	-913.2 kcal/mol	NIMAG = 1	-343.0 cm^{-1}
C	0.007806	0.410300	-0.000162
BR	2.773419	-0.739456	-0.000524
C	-0.460360	1.717304	0.000918
H	0.490869	2.684966	0.001873
H	-0.924722	2.075357	0.920714
H	-0.924399	2.076966	-0.918409
H	0.170621	-0.146090	0.914353
H	0.171142	-0.144368	-0.915628
BR	1.274114	4.263107	0.003405

3c E2-a-TS1	-920.2 kcal/mol	NIMAG = 1	-61.3 cm^{-1}
C	0.559551	0.549658	-0.003243
C	1.911204	0.672571	-0.001012
H	2.000714	2.350351	-0.001168
H	2.480365	0.570149	-0.922722
H	2.477127	0.571683	0.922842
H	-0.014396	0.546211	-0.922442
H	-0.017685	0.548033	0.913884
BR	-1.048195	-2.163709	0.000445
BR	2.430879	3.860790	0.000324

3c E2-a-INT

[c]

3c E2-a-TS2

[c]

3c E2-PC -939.7 kcal/mol NIMAG = 0

BR	-0.579040	-0.141062	0.000000
BR	2.893080	-0.141062	0.000000
C	1.823294	4.356431	0.000000
C	0.490746	4.356431	0.000000
H	2.393859	5.284875	0.000000
H	2.385728	3.425607	0.000000
H	-0.079818	5.284875	0.000000
H	-0.071688	3.425607	0.000000
H	1.157020	-0.140398	0.000000

3c S_N2-PC -942.1 kcal/mol NIMAG = 0

C	0.026712	0.013995	-0.874515
C	1.473959	0.009710	-0.452315
BR	-0.321923	0.036455	3.102441
BR	-0.201756	0.036704	-2.874372
H	-0.510631	0.897763	-0.534288
H	-0.510198	-0.878152	-0.555897
H	2.002080	0.902750	-0.801193
H	1.472812	0.002062	0.646230
H	2.001558	-0.877900	-0.815654

4c RC-a -934.9 kcal/mol NIMAG = 0

C	0.167904	0.459040	-0.000171
C	1.675031	0.470957	0.000119
BR	2.461179	2.322134	-0.000289
H	-0.242432	0.945002	0.890032
H	2.100761	0.008557	0.890804
H	2.101069	0.008021	-0.890152
H	-0.242102	0.944489	-0.890809
H	-0.150981	-0.594047	0.000099
I	-0.361821	-3.803343	0.002236

4c RC-s

[b]

4c S_N2-TS -921.7 kcal/mol NIMAG = 1 -254.8 cm^{-1}

C	-0.004226	0.030762	-0.000166
BR	2.631606	0.010322	-0.000348
C	-0.315871	1.497446	-0.000117
I	-2.594284	-1.014471	0.020485
H	0.122982	-0.514407	0.918169
H	0.114985	-0.511600	-0.921715
H	-0.843407	1.787542	0.912264
H	0.613485	2.072440	-0.058050

H	-0.938945	1.764980	-0.857627

4c E2-s-TS	-900.6 kcal/mol	NIMAG = 1	-141.5 cm^{-1}
C	0.140072	0.585992	-0.000438
BR	2.947640	-1.355329	-0.000971
C	-0.482318	1.821830	0.001384
H	0.404386	2.832861	0.002367
H	-0.977671	2.136393	0.920699
H	-0.978036	2.138920	-0.916862
H	0.436302	0.075250	0.910013
H	0.436065	0.077981	-0.912484
I	1.169637	4.648129	0.003696

4c E2-a-TS1	-905.2 kcal/mol	NIMAG = 1	-35.8 cm^{-1}
C	0.786041	0.404502	-0.002819
C	2.119391	0.607118	-0.001211
H	2.110238	2.472539	-0.000685
H	2.694906	0.590925	-0.924424
H	2.692690	0.590936	0.923362
H	0.212425	0.324768	-0.919992
H	0.210012	0.324623	0.912816
BR	-1.236759	-2.398675	0.000411
I	2.519904	4.141222	0.000596

4c E2-a-INT	-906.9 kcal/mol	NIMAG = 0	
C	0.533964	-0.385612	0.640595
C	1.846824	-0.319592	0.406364
H	-0.192177	-0.597026	-0.148455
H	0.142694	-0.235995	1.647495
H	2.244019	-0.476344	-0.595255
H	2.572312	-0.121022	1.194985
H	1.241249	2.347842	0.275556
I	1.489228	3.955260	0.393705
BR	-2.033378	-1.236060	-2.149591

4c E2-a-TS2
[c]

4c E2-PC	-928.0 kcal/mol	NIMAG = 0	
C	0.455396	1.623323	-0.013769
C	1.785541	1.549545	0.012060
H	2.407445	2.444118	0.015696
H	2.296162	0.589443	0.029914
H	-0.160588	0.727194	-0.017355
H	-0.061875	2.582028	-0.032348
H	0.919036	-3.074721	-0.000466
BR	2.542250	-3.085755	-0.005700
I	-1.160062	-3.064746	0.005942

4c S_N2-PC	-929.1 kcal/mol	NIMAG = 0	
C	-0.078268	0.254247	-0.000201
C	1.425057	0.190538	0.000150

I	2.418946	2.176482	-0.000105
H	-0.468882	0.756853	0.890011
H	1.833363	-0.285607	0.890130
H	1.833758	-0.286030	-0.889428
H	-0.468487	0.756445	-0.890823
H	-0.430983	-0.791451	-0.000023
BR	-0.372289	-3.620042	0.002070

5c RC-a	-928.6 kcal/mol	NIMAG = 0	
C	1.404806	0.082679	0.028043
BR	3.393690	0.330805	-0.002686
C	0.651258	1.389541	0.007942
AT	-3.038952	-1.129275	0.018827
H	1.223871	-0.496324	0.933124
H	1.200461	-0.536348	-0.844997
H	0.882653	2.004925	0.882615
H	0.864706	1.966473	-0.896923
H	-0.419193	1.145054	0.022727

5c RC-s
[b]

5c S_N2-TS	-915.4 kcal/mol	NIMAG = 1	-240.6 cm^{-1}
C	0.046028	0.059904	-0.000447
BR	2.678850	0.021334	-0.000538
C	-0.260448	1.528023	-0.000523
AT	-2.619339	-1.022792	0.020634
H	0.166753	-0.486373	0.918227
H	0.159115	-0.484000	-0.921952
H	-0.789467	1.820890	0.910160
H	0.671056	2.100511	-0.054840
H	-0.879109	1.800450	-0.859773

5c E2-s-TS	-893.5 kcal/mol	NIMAG = 1	-39.1 cm^{-1}
C	0.391531	0.641214	-0.023908
BR	3.019968	-1.764089	0.000433
C	-0.299242	1.847281	0.015728
H	0.520385	2.855994	0.004588
H	-0.799882	2.102289	0.951043
H	-0.853991	2.130921	-0.879980
H	0.751430	0.134788	0.866485
H	0.696516	0.170772	-0.953676
AT	1.249836	4.836826	0.003832

5c E2-a-TS1	-896.7 kcal/mol	NIMAG = 1	-34.4 cm^{-1}
C	0.866570	0.304054	0.001342
C	2.194523	0.527454	-0.001318
H	2.152669	2.469554	0.000492
H	2.766720	0.534368	-0.926486
H	2.770716	0.532561	0.921376
H	0.290968	0.209182	-0.913354
H	0.295054	0.207443	0.918432

BR	-1.315161	-2.498317	-0.000012
AT	2.551115	4.223002	0.000401

5c E2-a-INT -898.4 kcal/mol NIMAG = 0

C	0.354925	-0.721911	0.519797
C	1.667106	-0.641305	0.289970
H	-0.365173	-0.955683	-0.267582
H	-0.043209	-0.559142	1.521914
H	2.071705	-0.808199	-0.706998
H	2.385471	-0.415808	1.077910
H	1.057839	2.253283	0.123167
AT	1.350925	3.941133	0.272388
BR	-2.242299	-1.631801	-2.293140

5c E2-a-TS2

[c]

5c E2-PC -920.5 kcal/mol NIMAG = 0

C	0.085488	1.610168	-0.008320
C	1.416575	1.666351	0.009065
H	1.946911	2.618048	0.009511
H	2.020575	0.761939	0.022860
H	-0.438767	0.657309	-0.008949
H	-0.524273	2.512921	-0.022854
H	0.706755	-3.061872	0.000938
AT	2.906030	-3.141546	-0.006332
BR	-0.901646	-3.005528	0.004943

5c S_N2-PC -921.7 kcal/mol NIMAG = 0

C	-0.596821	-0.132036	0.004930
BR	3.187026	0.092474	-0.002849
C	-0.589125	1.375077	0.002133
AT	-2.748523	-1.026765	0.020960
H	-0.144082	-0.570370	0.891080
H	-0.156656	-0.573678	-0.885917
H	-1.067199	1.793099	0.893620
H	0.467014	1.676213	-0.004513
H	-1.077978	1.789924	-0.884988

1d RC-a

[a]

1d RC-s

[a]

1d S_N2-TS

[a]

1d E2-s-TS -954.2 kcal/mol NIMAG = 1 -159.6 cm^{-1}

C	2.064044	-0.056602	-0.265384
C	2.419517	1.275277	0.249962

H	1.641918	2.196104	0.076071
H	3.276492	1.649001	-0.332212
H	2.685968	1.247109	1.313344
H	2.123446	-0.162375	-1.348753
H	2.474876	-0.933743	0.231992
F	0.948063	3.400302	-0.113923
I	-0.178300	-0.725427	0.018003

1d E2-a-TS1

[a]

1d E2-a-INT

[a]

1d E2-a-TS2

[a]

1d E2-PC	-988.1 kcal/mol		NIMAG = 0
C	0.511578	1.527640	-0.010298
C	1.822826	1.291487	0.007183
H	2.550416	2.102561	0.014466
H	2.210628	0.275506	0.014803
H	-0.206396	0.710519	-0.017161
H	0.115779	2.542959	-0.018167
H	1.455846	-2.921652	-0.002356
F	2.421473	-2.847246	-0.005072
I	-0.927933	-3.075599	0.005106

1d S_N2-PC	-988.7 kcal/mol		NIMAG = 0
C	0.479820	0.014523	-0.000231
F	1.905362	0.003275	-0.000002
C	-0.059390	1.422308	-0.000189
H	-1.155588	1.374150	-0.000365
H	0.267736	1.968661	-0.891628
H	0.267440	1.968518	0.891441
H	0.164589	-0.539297	-0.890597
H	0.164330	-0.539417	0.889967
I	-4.303862	0.265175	0.001049

2d RC-a	-935.2 kcal/mol		NIMAG = 0
C	-0.477406	-0.011449	0.007668
CL	3.051918	0.094961	-0.023685
C	-0.411342	1.494885	0.006641
I	-2.573308	-0.784510	0.012120
H	-0.037707	-0.463514	0.893310
H	-0.040293	-0.464431	-0.878746
H	-0.877218	1.929063	0.897227
H	0.658477	1.743250	0.002883
H	-0.882386	1.927822	-0.881821

2d RC-s

[b]

2d S_N2-TS	-929.6 kcal/mol	NIMAG = 1	-276.2 cm^{-1}
C	0.046900	0.127200	-0.008300
CL	2.580100	0.053400	-0.017800
C	-0.188800	1.607200	0.006200
I	-2.520600	-0.800400	0.012300
H	0.196000	-0.422600	0.903400
H	0.180400	-0.402300	-0.934800
H	-0.693300	1.919200	0.924500
H	0.776200	2.120300	-0.052400
H	-0.800100	1.917900	-0.845400

2d E2-s-TS	-915.9 kcal/mol	NIMAG = 1	-409.2 cm^{-1}
C	0.038003	-0.133108	-0.000032
I	2.941354	0.052376	-0.000001
C	-0.833096	0.967647	0.000023
H	-0.329354	2.186088	-0.000108
H	-1.408211	1.109968	0.917115
H	-1.408472	1.109895	-0.916913
H	0.284631	-0.656337	0.914783
H	0.284493	-0.656353	-0.914875
CL	-0.172633	3.838751	-0.000267

2d E2-a-TS1	-925.7 kcal/mol	NIMAG = 1	-372.4 cm^{-1}
C	-0.133565	0.052450	0.010004
C	1.243445	0.158188	0.005752
H	1.477454	1.584326	0.011507
H	1.781630	-0.067661	-0.914003
H	1.787929	-0.076127	0.919666
H	-0.703450	0.161811	-0.905323
H	-0.697123	0.153308	0.930214
I	-1.397523	-2.665585	0.001731
CL	2.023532	3.034453	0.016228

2d E2-a-INT

[c]

2d E2-a-TS2

[c]

2d E2-PC	-941.6 kcal/mol	NIMAG = 0
C	0.099671	1.564038	-0.006114
C	1.426826	1.446162	0.005890
H	2.073936	2.322600	0.008028
H	1.913076	0.473573	0.013573
H	-0.542474	0.686307	-0.008379
H	-0.390112	2.537194	-0.014382
H	0.917506	-3.103809	0.000284
I	3.157329	-3.162537	-0.006929
CL	-0.481345	-3.062990	0.003461

2d S_N2-PC -948.4 kcal/mol NIMAG = 0

C	0.409161	0.149722	0.057444
CL	2.209345	-0.173372	-0.071570
C	0.065085	1.617468	-0.033222
H	-1.025417	1.707343	0.053818
H	0.378795	2.043115	-0.991372
H	0.531663	2.188091	0.775676
H	-0.043948	-0.427310	-0.749099
H	0.108142	-0.282839	1.011933
I	-4.161471	0.763362	0.407329

3d RC-a -929.1 kcal/mol NIMAG = 0

C	-0.078268	0.254247	-0.000201
C	1.425057	0.190538	0.000150
I	2.418946	2.176482	-0.000105
H	-0.468882	0.756853	0.890011
H	1.833363	-0.285607	0.890130
H	1.833758	-0.286030	-0.889428
H	-0.468487	0.756445	-0.890823
H	-0.430983	-0.791451	-0.000023
BR	-0.372289	-3.620042	0.002070

3d RC-s

[b]

3d S_N2-TS -921.7 kcal/mol NIMAG = 1 -254.8 cm^{-1}

C	-0.004226	0.030762	-0.000166
BR	2.631606	0.010322	-0.000348
C	-0.315871	1.497446	-0.000117
I	-2.594284	-1.014471	0.020485
H	0.122982	-0.514407	0.918169
H	0.114985	-0.511600	-0.921715
H	-0.843407	1.787542	0.912264
H	0.613485	2.072440	-0.058050
H	-0.938945	1.764980	-0.857627

3d E2-s-TS -905.2 kcal/mol NIMAG = 1 -236.6 cm^{-1}

C	-0.124182	0.469973	-0.000311
I	2.799561	-0.795775	-0.000552
C	-0.563937	1.792610	0.001105
H	0.372797	2.725158	0.001926
H	-1.037519	2.143152	0.919620
H	-1.037640	2.145073	-0.916611
H	0.018470	-0.090650	0.915167
H	0.018630	-0.088604	-0.917021
BR	1.179279	4.334045	0.003435

3d E2-a-TS1 -912.7 kcal/mol NIMAG = 1 -143.9 cm^{-1}

C	-0.740610	0.006079	0.027570
I	2.561154	-0.014850	-0.007596
C	-1.474365	1.155060	0.021359

H	-2.951985	0.579897	0.054970
BR	-4.543858	0.338302	0.086698
H	-1.619897	1.704781	-0.906422
H	-1.591553	1.732652	0.936107
H	-0.505374	-0.525185	-0.887527
H	-0.478337	-0.498153	0.950477

3d E2-a-INT

[c]

3d E2-a-TS2

[c]

3d E2-PC -928.0 kcal/mol NIMAG = 0

C	0.455396	1.623323	-0.013769
C	1.785541	1.549545	0.012060
H	2.407445	2.444118	0.015696
H	2.296162	0.589443	0.029914
H	-0.160588	0.727194	-0.017355
H	-0.061875	2.582028	-0.032348
H	0.919036	-3.074721	-0.000466
BR	2.542250	-3.085755	-0.005700
I	-1.160062	-3.064746	0.005942

3d S_N2-PC -934.9 kcal/mol NIMAG = 0

C	0.167904	0.459040	-0.000171
C	1.675031	0.470957	0.000119
BR	2.461179	2.322134	-0.000289
H	-0.242432	0.945002	0.890032
H	2.100761	0.008557	0.890804
H	2.101069	0.008021	-0.890152
H	-0.242102	0.944489	-0.890809
H	-0.150981	-0.594047	0.000099
I	-0.361821	-3.803343	0.002236

4d RC-a -922.2 kcal/mol NIMAG = 0

I	-0.005839	-0.061466	3.264795
C	0.062065	0.425913	-0.922645
C	0.240725	1.888445	-0.594545
I	0.004340	0.026208	-3.100716
H	-0.879735	0.017008	-0.562019
H	0.882209	-0.196463	-0.570075
H	-0.582914	2.498587	-0.977790
H	0.254775	1.966376	0.499986
H	1.182622	2.284983	-0.985888

4d RC-s

[b]

4d S_N2-TS -912.4 kcal/mol NIMAG = 1 -236.0 cm^{-1}

I	-0.005389	-0.032306	2.759219
C	0.118658	0.538822	-0.000004

C	0.264141	2.031935	0.000008
I	-0.005388	-0.032306	-2.759219
H	-0.842717	0.058415	0.000002
H	0.990429	-0.094312	-0.000010
H	-0.710043	2.526985	0.000056
H	0.817391	2.361331	0.884220
H	0.817286	2.361332	-0.884273

4d E2-s-TS -893.2 kcal/mol NIMAG = 1 -103.4 cm^{-1}

C	-0.016543	0.862242	0.000721
I	2.923094	-1.231415	-0.000968
C	-0.553441	2.142220	0.000859
H	0.370841	3.086351	0.002166
H	-1.039869	2.476576	0.918356
H	-1.038302	2.477419	-0.917159
H	0.235974	0.334988	0.914751
H	0.237443	0.335687	-0.913281
I	1.236130	4.886013	0.003925

4d E2-a-TS1 -898.1 kcal/mol NIMAG = 1 -53.6 cm^{-1}

C	-0.874062	0.271831	0.051315
I	2.704836	-0.050304	-0.011994
C	-1.646392	1.381330	0.047681
H	-3.249128	0.619304	0.069628
I	-4.952015	0.257292	0.087064
H	-1.872518	1.904035	-0.879043
H	-1.854180	1.923197	0.967686
H	-0.572086	-0.223739	-0.864975
H	-0.553954	-0.204407	0.971780

4d E2-a-INT -900.2 kcal/mol NIMAG = 0

C	-0.028513	-0.368604	-0.093350
C	1.211254	-0.728008	0.244528
H	1.695056	2.052093	-0.206715
H	1.883359	-1.178849	-0.483478
H	1.599720	-0.598440	1.254091
H	-0.423379	-0.508100	-1.100032
H	-0.700811	0.075160	0.640781
I	2.422844	3.492086	0.002460
I	-1.716945	-0.998559	-3.951779

4d E2-a-TS2 -900.2 kcal/mol NIMAG = 1 -18.4 cm^{-1}

H	-0.110505	0.149427	0.495800
I	3.026675	0.115621	0.428017
C	-1.921983	1.202612	0.132728
C	-1.200787	0.138438	0.488420
H	-1.559625	-0.469469	-2.447042
H	-1.440741	2.133182	-0.163335
H	-3.011445	1.191538	0.126064
H	-1.686164	-0.790303	0.788339
I	-2.115672	-1.040398	-3.862601

4d E2-PC	-915.3 kcal/mol		NIMAG = 0
C	-0.000001	-0.665881	-2.250356
C	-0.000002	0.666381	-2.240099
H	-0.000001	-1.229468	-3.182560
H	0.000000	-1.238371	-1.325864
H	-0.000002	1.244064	-3.163627
H	-0.000002	1.224810	-1.307043
H	0.000000	0.004556	2.641601
I	0.000001	-1.934478	2.645403
I	-0.000002	1.944386	2.642750

4d S_N2-PC	-922.2 kcal/mol		NIMAG = 0
I	-0.005839	-0.061466	3.264795
C	0.062065	0.425913	-0.922645
C	0.240725	1.888445	-0.594545
I	0.004340	0.026208	-3.100716
H	-0.879735	0.017008	-0.562019
H	0.882209	-0.196463	-0.570075
H	-0.582914	2.498587	-0.977790
H	0.254775	1.966376	0.499986
H	1.182622	2.284983	-0.985888

5d RC-a	-915.7 kcal/mol		NIMAG = 0
C	-1.064848	-0.359424	-0.005547
AT	3.394549	0.068507	-0.001942
C	-0.920309	1.142913	-0.001216
I	-3.170485	-1.037614	0.024237
H	-0.632767	-0.833283	0.874114
H	-0.661348	-0.825731	-0.902666
H	-1.353957	1.593884	0.896451
H	0.156521	1.358805	-0.014736
H	-1.379438	1.601789	-0.882149

5d RC-s
[b]

5d S_N2-TS	-906.2 kcal/mol		NIMAG = 1	-230.2 cm^{-1}
C	0.008958	0.074630	0.048532	
At	2.913469	0.022474	-0.175585	
C	-0.276442	1.546998	-0.004765	
I	-2.562845	-1.047585	0.167493	
H	0.174115	-0.415911	0.993625	
H	0.084561	-0.524285	-0.840873	
H	-1.230399	1.769424	0.482855	
H	0.503669	2.110045	0.515947	
H	-0.327515	1.905408	-1.035828	

5d E2-s-TS	-886.2 kcal/mol		NIMAG = 1	-25.3 cm^{-1}
C	0.146565	0.910553	-0.001484	
I	2.998528	-1.561254	-0.001133	
C	-0.480669	2.153713	0.002643	
H	0.373587	3.124254	0.002626	

H	-0.997275	2.440577	0.919961
H	-1.001385	2.444614	-0.911064
H	0.450616	0.405747	0.910148
H	0.446744	0.410250	-0.916876
AT	1.175984	5.084185	0.004043

5d E2-a-TS1 -889.8 kcal/mol NIMAG = 1 -47.2 cm^{-1}

C	-0.973360	0.324690	0.047133
I	2.714268	-0.057534	-0.011795
C	-1.776895	1.409376	0.049175
H	-3.416538	0.599651	0.069891
AT	-5.194849	0.202492	0.089719
H	-2.027568	1.925401	-0.874829
H	-2.009187	1.935766	0.972154
H	-0.649604	-0.152831	-0.871584
H	-0.631865	-0.142493	0.964792

5d E2-a-INT -891.8 kcal/mol NIMAG = 0

C	*-0.658872*	*2.257149*	*0.656690*
I	*2.874854*	*-0.224428*	*-0.039991*
C	*-1.293676*	*2.931934*	*-0.302303*
H	*-3.719077*	*0.623594*	*0.045255*
AT	*-5.342836*	*0.073044*	*0.089619*
H	*-0.973290*	*2.868472*	*-1.340714*
H	*-2.147141*	*3.575809*	*-0.092278*
H	*0.200218*	*1.620003*	*0.450133*
H	*-0.978445*	*2.327715*	*1.696311*

5d E2-a-TS2

[c]

5d E2-PC -907.8 kcal/mol NIMAG = 0

C	0.302004	1.766983	-0.009580
C	1.634178	1.773509	0.008819
H	2.200115	2.704310	0.010507
H	2.204488	0.847595	0.022280
H	-0.259323	0.835583	-0.011402
H	-0.273081	2.692028	-0.023685
H	0.886159	-3.091189	-0.000178
AT	2.950061	-3.116800	-0.006471
I	-1.023874	-3.075410	0.005346

5d S_N2-PC -914.6 kcal/mol NIMAG = 0

C	0.830981	0.133166	-0.011275
AT	3.150722	0.072904	-0.002746
C	0.278654	1.536551	-0.016706
I	-3.102195	-1.143569	0.027946
H	0.567488	-0.435643	0.877818
H	0.574916	-0.439686	-0.900010
H	-0.815572	1.446253	-0.018640
H	0.577812	2.101359	0.871587
H	0.582361	2.096553	-0.906520

1e RC-a

[a]

1e RC-s

[a]

1e S_N2-TS

[a]

1e E2-s-TS -938.8 kcal/mol NIMAG = 1 -55.1 cm^{-1}

C	0.282812	0.128722	-0.319086
AT	2.583695	-0.515214	0.024094
C	-0.121134	1.403852	0.313255
H	0.578230	2.344872	0.125036
H	-0.310114	1.285195	1.387276
H	-1.052879	1.736716	-0.174725
H	-0.142128	-0.796819	0.068415
H	0.262170	0.137712	-1.409598
F	1.236537	3.640648	-0.134228

1e E2-a-TS1

[a]

1e E2-a-INT

[a]

1e E2-a-TS2

[a]

1e E2-PC -980.5 kcal/mol NIMAG = 0

C	0.511578	1.527640	-0.010298
C	1.822826	1.291487	0.007183
H	2.550416	2.102561	0.014466
H	2.210628	0.275506	0.014803
H	-0.206396	0.710519	-0.017161
H	0.115779	2.542959	-0.018167
H	1.455846	-2.921652	-0.002356
F	2.421473	-2.847246	-0.005072
I	-0.927933	-3.075599	0.005106

1e S_N2-PC -982.3 kcal/mol NIMAG = 0

C	0.700636	2.356187	0.010669
C	1.843346	1.375056	0.086743
H	2.513012	1.484334	-0.773265
H	1.430050	0.358966	0.082126
H	2.421034	1.514284	1.007008
H	0.117935	2.227894	-0.907265
H	0.029289	2.262829	0.870497
F	1.172405	3.700426	0.007796
AT	-0.889227	-2.268795	-0.010456

2e RC-a	-927.7 kcal/mol	NIMAG = 0	
C	0.058156	0.279508	-0.002961
C	1.548725	0.061416	0.008437
AT	-0.515649	2.550644	0.003086
CL	-0.319263	-3.175966	-0.020889
H	-0.450182	-0.101576	0.878816
H	-0.435334	-0.092712	-0.896827
H	2.036906	0.486704	-0.874362
H	2.021684	0.476744	0.904158
H	1.691644	-1.027426	0.003652

2e RC-s
[b]

2e S_N2-TS	-923.2 kcal/mol	NIMAG = 1	-265.8 cm^{-1}
C	0.107034	-0.219922	-0.010999
C	1.604995	-0.173811	0.008012
AT	-0.526750	2.521403	0.003016
CL	-0.294671	-2.724046	-0.017006
H	-0.460016	-0.294741	0.899433
H	-0.432524	-0.285331	-0.939182
H	1.995546	0.388762	-0.844355
H	1.977140	0.289386	0.925784
H	1.992434	-1.196379	-0.045551

2e E2-s-TS	-910.0 kcal/mol	NIMAG = 1	-471.3 cm^{-1}
C	-0.068055	0.310830	-0.000219
AT	2.763517	-0.703692	-0.000493
C	-0.439397	1.663061	0.000823
H	0.513358	2.592366	0.001844
H	-0.907886	2.023709	0.918539
H	-0.907507	2.025263	-0.916466
H	-0.038139	-0.267400	0.914569
H	-0.037765	-0.265890	-0.915955
CL	1.318381	4.024114	0.003220

2e E2-a-TS1	-919.5 kcal/mol	NIMAG = 1	-360.9 cm^{-1}
C	-0.098021	0.103644	0.010094
C	1.278456	0.204103	0.005829
H	1.518584	1.648549	0.011643
H	1.817790	-0.017189	-0.914155
H	1.824077	-0.025647	0.920021
H	-0.668075	0.212477	-0.905513
H	-0.661763	0.203980	0.930564
AT	-1.414085	-2.691158	0.001672
CL	2.054118	3.086255	0.016370

2e E2-a-INT
[c]

2e E2-a-TS2

[c]

2e E2-PC -934.2 kcal/mol NIMAG = 0

C	0.722356	1.619723	-0.013832
C	2.051556	1.530558	0.010143
H	2.684416	2.417404	0.015978
H	2.550154	0.564136	0.024456
H	0.094760	0.731401	-0.019365
H	0.216941	2.584779	-0.028585
H	1.215532	-2.948149	0.002295
CL	2.607460	-2.881010	-0.006315
AT	-1.140854	-3.057081	0.005751

2e S_N2-PC -942.0 kcal/mol NIMAG = 0

C	0.677947	0.068952	-0.015623
CL	2.507884	0.028068	-0.097218
C	0.116984	1.470350	-0.075009
H	-0.976067	1.395097	-0.008554
H	0.381798	1.968155	-1.012954
H	0.475137	2.079220	0.760604
H	0.340978	-0.543330	-0.852822
H	0.419605	-0.435220	0.915957
AT	-4.055141	-0.018132	0.510387

3e RC-a -921.7 kcal/mol NIMAG = 0

C	-0.596821	-0.132036	0.004930
BR	3.187026	0.092474	-0.002849
C	-0.589125	1.375077	0.002133
AT	-2.748523	-1.026765	0.020960
H	-0.144082	-0.570370	0.891080
H	-0.156656	-0.573678	-0.885917
H	-1.067199	1.793099	0.893620
H	0.467014	1.676213	-0.004513
H	-1.077978	1.789924	-0.884988

3e RC-s

[b]

3e S_N2-TS -915.4 kcal/mol NIMAG = 1 -240.6 cm^{-1}

C	0.046028	0.059904	-0.000447
BR	2.678850	0.021334	-0.000538
C	-0.260448	1.528023	-0.000523
AT	-2.619339	-1.022792	0.020634
H	0.166753	-0.486373	0.918227
H	0.159115	-0.484000	-0.921952
H	-0.789467	1.820890	0.910160
H	0.671056	2.100511	-0.054840
H	-0.879109	1.800450	-0.859773

3e E2-s-TS -899.3 kcal/mol NIMAG = 1 -338.0 cm^{-1}

C	-0.190335	0.534675	-0.000010

AT	2.823225	-0.828888	-0.000588
C	-0.624899	1.856189	0.000920
H	0.334517	2.794975	0.001934
H	-1.088641	2.217428	0.919966
H	-1.088141	2.218866	-0.917815
H	-0.042243	-0.024244	0.916137
H	-0.041807	-0.022814	-0.916956
BR	1.163888	4.363315	0.003473

3e E2-a-TS1 -906.5 kcal/mol NIMAG = 1 -138.0 cm^{-1}

C	0.147611	0.215068	0.009111
C	1.506209	0.327795	0.005343
H	1.689266	1.920732	0.012416
H	2.063735	0.188580	-0.918626
H	2.069622	0.179390	0.924322
H	-0.429259	0.241224	-0.908450
H	-0.423416	0.232088	0.930487
AT	-1.472183	-2.766792	0.001556
BR	2.207731	3.438456	0.017641

3e E2-a-INT

[c]

3e E2-a-TS2

[c]

3e E2-PC -920.5 kcal/mol NIMAG = 0

C	0.085488	1.610168	-0.008320
C	1.416575	1.666351	0.009065
H	1.946911	2.618048	0.009511
H	2.020575	0.761939	0.022860
H	-0.438767	0.657309	-0.008949
H	-0.524273	2.512921	-0.022854
H	0.706755	-3.061872	0.000938
AT	2.906030	-3.141546	-0.006332
BR	-0.901646	-3.005528	0.004943

3e S_N2-PC -928.6 kcal/mol NIMAG = 0

C	1.404806	0.082679	0.028043
BR	3.393690	0.330805	-0.002686
C	0.651258	1.389541	0.007942
AT	-3.038952	-1.129275	0.018827
H	1.223871	-0.496324	0.933124
H	1.200461	-0.536348	-0.844997
H	0.882653	2.004925	0.882615
H	0.864706	1.966473	-0.896923
H	-0.419193	1.145054	0.022727

4e RC-a -914.6 kcal/mol NIMAG = 0

C	0.830981	0.133166	-0.011275
AT	3.150722	0.072904	-0.002746
C	0.278654	1.536551	-0.016706

I	-3.102195	-1.143569	0.027946
H	0.567488	-0.435643	0.877818
H	0.574916	-0.439686	-0.900010
H	-0.815572	1.446253	-0.018640
H	0.577812	2.101359	0.871587
H	0.582361	2.096553	-0.906520

4e RC-s

[b]

4e S_N2-TS -906.2 kcal/mol NIMAG = 1 -230.2 cm^{-1}

C	0.008958	0.074630	0.048532
At	2.913469	0.022474	-0.175585
C	-0.276442	1.546998	-0.004765
I	-2.562845	-1.047585	0.167493
H	0.174115	-0.415911	0.993625
H	0.084561	-0.524285	-0.840873
H	-1.230399	1.769424	0.482855
H	0.503669	2.110045	0.515947
H	-0.327515	1.905408	-1.035828

4e E2-s-TS -887.1 kcal/mol NIMAG = 1 -179.4 cm^{-1}

C	-0.105466	1.001882	0.001082
AT	2.958411	-1.234103	-0.000926
C	-0.689113	2.258194	0.000631
H	0.219323	3.243789	0.002091
H	-1.180221	2.583834	0.918446
H	-1.178031	2.584194	-0.918228
H	0.169369	0.487202	0.916291
H	0.171397	0.487315	-0.913573
I	1.047078	5.033684	0.004055

4e E2-a-TS1 -891.9 kcal/mol NIMAG = 1 -59.4 cm^{-1}

C	0.451885	0.190038	-0.009077
C	1.794478	0.354836	-0.001338
H	1.885209	2.121349	0.008211
H	2.371382	0.298800	-0.921824
H	2.361376	0.291238	0.924820
H	-0.117505	0.148907	-0.931523
H	-0.127694	0.141521	0.906528
AT	-1.573435	-2.902474	0.002052
I	2.369145	3.796137	0.020792

4e E2-a-INT

[c]

4e E2-a-TS2

[c]

4e E2-PC -907.8 kcal/mol NIMAG = 0

C	0.302004	1.766983	-0.009580
C	1.634178	1.773509	0.008819

H	2.200115	2.704310	0.010507
H	2.204488	0.847595	0.022280
H	-0.259323	0.835583	-0.011402
H	-0.273081	2.692028	-0.023685
H	0.886159	-3.091189	-0.000178
AT	2.950061	-3.116800	-0.006471
I	-1.023874	-3.075410	0.005346

4e S_N2-PC	-915.7 kcal/mol		NIMAG = 0
C	-1.064848	-0.359424	-0.005547
AT	3.394549	0.068507	-0.001942
C	-0.920309	1.142913	-0.001216
I	-3.170485	-1.037614	0.024237
H	-0.632767	-0.833283	0.874114
H	-0.661348	-0.825731	-0.902666
H	-1.353957	1.593884	0.896451
H	0.156521	1.358805	-0.014736
H	-1.379438	1.601789	-0.882149

5e RC-a	-908.2 kcal/mol		NIMAG = 0
C	0.002624	0.243005	-0.924916
AT	0.069159	-0.264749	-3.188687
C	-0.192817	1.715356	-0.659239
AT	0.075952	-0.322536	3.292741
H	0.951265	-0.144382	-0.559123
H	-0.811537	-0.378720	-0.558301
H	0.623392	2.319737	-1.066659
H	-1.138925	2.085585	-1.065712
H	-0.209700	1.846354	0.430435

5e RC-s
[b]

5e S_N2-TS	-900.0 kcal/mol		NIMAG = 1	-221.8 cm^{-1}
C	-0.060691	0.317538	0.000001	
AT	0.075155	-0.299470	-2.832165	
C	-0.204547	1.811894	-0.000007	
AT	0.075155	-0.299471	2.832166	
H	0.900933	-0.162892	-0.000005	
H	-0.932848	-0.315433	0.000003	
H	0.770503	2.305131	-0.000104	
H	-0.757098	2.146741	-0.883150	
H	-0.756959	2.146784	0.883206	

5e E2-s-TS	-880.1 kcal/mol		NIMAG = 1	-26.2 cm^{-1}
C	0.195225	1.267826	0.001642	
AT	2.952503	-1.617821	-0.001343	
C	-0.440590	2.506480	-0.000867	
H	0.416682	3.476713	0.000159	
H	-0.961378	2.795630	0.913453	
H	-0.958994	2.793159	-0.917221	
H	0.502834	0.772200	0.917348	

H	0.502502	0.767934	-0.912020
AT	1.249786	5.420566	0.004520

5e E2-a-TS1   -883.6 kcal/mol   NIMAG = 1   -39.5 cm^{-1}

C	0.964870	0.651423	-0.014451
C	2.305864	0.802264	-0.014459
H	2.434496	2.649255	-0.006717
H	2.877431	0.757887	-0.938591
H	2.878269	0.748243	0.908648
H	0.389769	0.606896	-0.933515
H	0.390503	0.596494	0.904390
AT	2.976942	4.383751	-0.001367
AT	-1.223195	-2.474343	0.001316

5e E2-a-INT   -885.5 kcal/mol   NIMAG = 0

C	2.652127	-0.631571	0.532841
C	3.599419	-0.382221	-0.371432
H	2.623976	2.980179	-0.051042
H	3.456479	-0.629574	-1.421810
H	4.553047	0.070135	-0.102175
H	1.699455	-1.089464	0.272623
H	2.801024	-0.387730	1.584141
AT	2.972894	4.660306	-0.001328
AT	-1.419059	-2.588710	-0.011267

5e E2-a-TS2

[c]

5e E2-PC   -900.5 kcal/mol   NIMAG = 0

H	-0.010978	-0.092665	-0.000001
AT	2.021170	-0.102923	-0.000001
AT	-2.042798	-0.091843	-0.000001
H	-1.232453	5.842318	-0.000001
H	-1.264476	3.986350	-0.000001
H	1.240517	5.793721	-0.000001
H	1.200040	3.938070	-0.000001
C	0.651873	4.877247	-0.000001
C	-0.680109	4.903512	-0.000001

5e S_N2-PC   -908.2 kcal/mol   NIMAG = 0

C	0.002624	0.243005	-0.924916
AT	0.069159	-0.264749	-3.188687
C	-0.192817	1.715356	-0.659239
AT	0.075952	-0.322536	3.292741
H	0.951265	-0.144382	-0.559123
H	-0.811537	-0.378720	-0.558301
H	0.623392	2.319737	-1.066659
H	-1.138925	2.085585	-1.065712
H	-0.209700	1.846354	0.430435

Table S2. Cartesian coordinates (in Å) of all reactants, CH_3–CH_2Y rotational transition states and products, computed at ZORA-OLYP/TZ2P.

F-	-86.8 kcal/mol
Cl-	-83.9 kcal/mol
Br-	-80.2 kcal/mol
I-	-74.6 kcal/mol
At-	-68.8 kcal/mol

st-C2H5F	-908.6 kcal/mol		NIMAG = 0
F	1.91218	-1.22708	0.00000
C	1.43209	0.09559	0.00000
C	-0.08089	0.11473	0.00000
H	-0.43227	1.15314	0.00000
H	-0.47956	-0.38219	0.88953
H	-0.47956	-0.38219	-0.88953
H	1.84380	0.58242	0.89214
H	1.84380	0.58242	-0.89214

ecl-C2H5F -	905.4 kcal/mol		NIMAG = 1	-268.9 cm^{-1}
F	1.94144	-1.22723	0.00000	
C	1.42264	0.08233	0.00000	
C	-0.10664	0.06461	0.00000	
H	-0.45446	-0.97220	0.00000	
H	-0.51932	0.55455	0.88657	
H	-0.51932	0.55455	-0.88657	
H	1.82893	0.57435	0.89019	
H	1.82893	0.57435	-0.89019	

st-C2H5Cl	-867.4 kcal/mol		NIMAG = 0
CL	-0.09137	-0.03508	0.00000
C	1.71675	0.03985	0.00000
C	2.23626	1.46429	0.00000
H	3.33319	1.44578	0.00000
H	1.90329	2.00920	0.88724
H	1.90329	2.00920	-0.88724
H	2.03582	-0.50925	0.88811
H	2.03582	-0.50925	-0.88811

ecl-C2H5Cl	-864.0 kcal/mol		NIMAG = 1	-242.2 cm^{-1}
CL	-0.09415	-0.04054	0.00000	
C	1.71481	0.04853	0.00000	
C	2.25402	1.48373	0.00000	
H	1.44264	2.21381	0.00000	
H	2.86840	1.67065	0.88565	
H	2.86840	1.67065	-0.88565	
H	2.02658	-0.50749	0.88487	
H	2.02658	-0.50749	-0.88487	

st-C2H5Br -853.6 kcal/mol			NIMAG = 0
BR	1.920759	-0.010972	0.000000

C	-0.060661	0.033469	0.000000
C	-0.614763	1.443281	0.000000
H	-1.711887	1.392525	0.000000
H	-0.302459	1.999855	-0.887477
H	-0.302459	1.999855	0.887477
H	-0.348744	-0.527564	-0.890424
H	-0.348744	-0.527564	0.890424

ecl-C2H5Br	-850.3 kcal/mol	NIMAG = 1	-230.6 cm^{-1}
BR	1.924719	-0.015111	0.000000
C	-0.057842	0.049995	0.000000
C	-0.631963	1.469477	0.000000
H	0.151158	2.229858	0.000000
H	-1.252580	1.637283	-0.885666
H	-1.252580	1.637283	0.885666
H	-0.338686	-0.517760	-0.886978
H	-0.338686	-0.517760	0.886978

st-C2H5I	-840.4 kcal/mol	NIMAG = 0	
I	2.105244	-0.008523	-0.000001
C	-0.077085	0.043868	0.000008
C	-0.644488	1.448557	-0.000004
H	-1.741368	1.387145	-0.000058
H	-0.339513	2.010768	-0.886584
H	-0.339598	2.010728	0.886630
H	-0.353476	-0.522065	-0.889822
H	-0.353454	-0.522052	0.889852

ecl-C2H5I	-828.0 kcal/mol	NIMAG = 1	-567.1 cm^{-1}
I	2.113095	-0.021185	0.000000
C	-0.011688	0.165385	0.000000
C	-0.734697	1.478575	0.000000
H	-0.194988	2.415857	0.000000
H	-1.392535	1.398381	-0.826401
H	-1.392535	1.398381	0.826401
H	-0.359433	-0.370793	-0.837932
H	-0.359433	-0.370793	0.837932

st-C2H5At	-833.2 kcal/mol	NIMAG = 0	
AT	2.229991	-0.004566	0.000000
C	-0.054542	0.042407	0.000006
C	-0.631213	1.442032	0.000000
H	-1.728553	1.375636	-0.000040
H	-0.332596	2.008002	-0.886370
H	-0.332645	2.007995	0.886389
H	-0.318854	-0.526867	-0.891103
H	-0.318864	-0.526876	0.891104

ecl-C2H5At	-820.8 kcal/mol	NIMAG = 1	-565.8 cm^{-1}
AT	2.235634	-0.012375	0.000000
C	0.010640	0.169961	0.000000
C	-0.733795	1.469997	0.000000

H	-0.214325	2.419091	0.000000
H	-1.390329	1.379496	-0.826509
H	-1.390329	1.379496	0.826509
H	-0.322627	-0.374183	-0.839034
H	-0.322627	-0.374183	0.839034

C2H4	-715.2 kcal/mol	NIMAG = 0	
C	0.000000	0.665683	0.000000
C	0.000000	-0.665683	0.000000
H	0.925267	1.237412	0.000000
H	-0.925267	1.237412	0.000000
H	0.925267	-1.237412	0.000000
H	-0.925267	-1.237412	0.000000

FHF-	-316.6 kcal/mol	NIMAG = 0	
F	0.00000	0.00000	1.15704
H	0.00000	0.00000	0.00000
F	0.00000	0.00000	-1.15704

FHCl-	-288.1 kcal/mol	NIMAG = 0	
F	-0.328874	0.000002	0.000002
H	0.669703	0.000002	0.000002
CL	2.543348	0.000002	0.000002

FHBr-	-280.0 kcal/mol	NIMAG = 0	
F	-0.474448	0.000011	0.000016
H	0.504713	0.000007	0.000011
BR	2.623052	-0.000001	-0.000001

FHI-	-270.9 kcal/mol	NIMAG = 0	
F	-0.578775	-0.000001	-0.000012
H	0.388440	-0.000001	-0.000008
I	2.784821	0.000000	0.000001

FHAt-	-263.6 kcal/mol	NIMAG = 0	
F	-0.605694	0.000002	0.000007
H	0.357808	0.000002	0.000005
AT	2.888071	0.000000	0.000000

ClHCl-	-245.4 kcal/mol	NIMAG = 0	
CL	0.00000	0.00000	1.58145
H	0.00000	0.00000	0.00000
CL	0.00000	0.00000	-1.58145

ClHBr-	-234.9 kcal/mol	NIMAG = 0	
CL	3.043514	-0.000373	-0.000628
H	1.584577	-0.000168	-0.000305
BR	-0.312479	0.000098	0.000114

ClHI-	-224.6 kcal/mol	NIMAG = 0	
CL	3.358363	0.000000	-0.000001

H	1.959746	-0.000001	-0.000002
I	-0.284059	-0.000003	-0.000005

ClHAt-	-217.4 kcal/mol	NIMAG = 0	
CL	3.552985	0.000001	-0.000008
H	2.162058	-0.000005	-0.000004
AT	-0.205038	-0.000015	0.000005

BrHBr-	-222.6 kcal/mol	NIMAG = 0	
BR	1.735098	0.000000	0.000000
H	0.000000	0.000000	0.000000
BR	-1.735098	0.000000	0.000000

BrHI-	-211.1 kcal/mol	NIMAG = 0	
BR	-0.831741	0.000150	-0.000085
H	0.787816	0.000085	-0.000045
I	2.873240	0.000002	0.000006

BrHAt-	-203.8 kcal/mol	NIMAG = 0	
BR	-1.055404	0.000038	-0.000028
H	0.549310	0.000021	-0.000015
AT	2.752977	-0.000003	0.000002

IHI-	-198.5 kcal/mol	NIMAG = 0	
I	1.939022	0.000000	0.000000
H	0.000000	0.000000	0.000000
I	-1.939022	0.000000	0.000000

IHAt-	-191.1 kcal/mol	NIMAG = 0	
I	3.362032	0.000001	0.000011
H	1.453587	-0.000001	-0.000018
AT	-0.611146	-0.000003	-0.000049

AtHAt-	-183.9 kcal/mol	NIMAG = 0	
AT	2.031763	0.000000	0.000000
H	0.000000	0.000000	0.000000
AT	-2.031763	0.000000	0.000000

HF	-180.5 kcal/mol	NIMAG = 0	
H	0.00000	0.00000	-0.02588
F	0.00000	0.00000	0.90136

HCl	-135.9 kcal/mol	NIMAG = 0	
H	0.00000	0.00000	0.01163
CL	0.00000	0.00000	1.29967

HBr	-120.4 kcal/mol	NIMAG = 0	
H	0.035226	0.000000	0.000000
BR	1.461550	0.000000	0.000000

HI	-105.8 kcal/mol	NIMAG = 0	

H	0.035408	0.000000	0.000000
I	1.652719	0.000000	0.000000

Hat	-98.2 kcal/mol	NIMAG = 0	
H	0.078637	0.000000	0.000000
AT	1.789623	0.000000	0.000000

[C2H4-F]-	-816.9 kcal/mol	NIMAG = 0	
C	0.07364	0.85980	0.00000
C	0.26902	-0.46460	0.00000
H	0.88891	1.59052	0.00000
H	-0.93506	1.27962	0.00000
H	1.30543	-0.82482	0.00000
H	-0.55126	-1.25336	0.00000
F	-1.65401	-2.49159	0.00000

[C2H4-Cl]-	-803.5 kcal/mol	NIMAG = 0	
C	-0.48775	0.27167	0.00000
C	0.83030	0.47730	0.00000
H	-1.20659	1.09182	0.00000
H	-0.90222	-0.73589	0.00000
H	1.22868	1.49287	0.00000
H	1.56715	-0.33000	0.00000
CL	3.75252	-1.95804	0.00000

[C2H4-Br]-	-798.5 kcal/mol	NIMAG = 0	
C	-2.667589	1.017912	0.000000
C	-1.867892	-0.048974	0.000000
H	-3.754170	0.931058	0.000000
H	-2.259101	2.027251	0.000000
H	-2.285640	-1.056129	0.000000
H	-0.780234	0.028928	0.000000
BR	2.240627	-0.023784	0.000000

[C2H4-I]-	-792.1 kcal/mol	NIMAG = 0	
C	-3.291236	-0.297581	0.000000
C	-2.268516	0.557281	0.000000
H	-1.228552	0.234605	0.000000
H	-4.327612	0.038935	0.000000
H	-3.129244	-1.374198	0.000000
H	-2.438191	1.633561	0.000000
I	2.148862	-0.013635	0.000000

[C2H4-At]-	-785.91 kcal/mol	NIMAG = 0	
C	-3.305019	-0.296381	0.000000
C	-2.269390	0.542515	0.000000
H	-1.234828	0.203267	0.000000
H	-4.335682	0.057018	0.000000
H	-3.160594	-1.375373	0.000000
H	-2.420631	1.621340	0.000000
AT	2.279764	-0.007336	0.000000

Table S3. Cartesian coordinates of H_3CHN^-, H_3CO^-, or H_3CS^-, + $C_2H_5Cl^-$ intermediates and transition states, computed at ZORA-OLYP/TZ2P (RC = reactant complex; S_N2-TS = S_N2 transition state; E2-TS = E2 transition state; E2-PC = E2 product complex; S_N2-PC = S_N2 product complex). [a] Nonexistent.

H_3CHN^-	-675.03 kcal/mol		NIMAG = 0
N	0.785739	1.615819	-0.100699
H	1.335560	1.101257	0.602819
H	-0.416430	-0.173311	-0.629723
C	0.293951	0.647942	-1.012148
H	-0.311956	1.146736	-1.800120
H	1.041763	0.024037	-1.626088

H_3CHN^- RC
[a]

H_3CHN^- S_N2-TS		-X kcal/mol	NIMAG = 1	$-94.6 cm^{-1}$
C	-0.711862	-5.930841	0.243857	
C	-0.429699	-4.453813	0.184919	
Cl	1.568060	-4.210505	0.532435	
H	-0.511559	-6.336360	1.242144	
H	-0.090757	-6.478076	-0.474302	
H	-1.767162	-6.077107	-0.010280	
H	-0.851529	-3.848941	0.976477	
H	-0.536688	-3.990957	-0.786999	
N	-3.144978	-4.061730	-0.302113	
H	-3.349978	-4.057335	0.705699	
H	-3.313512	-2.701481	-1.878244	
C	-3.486983	-2.752941	-0.786376	
H	-4.559588	-2.436943	-0.659312	
H	-2.905659	-1.881381	-0.367721	

H_3CHN^- E2-TS
[a]

H_3CHN^- S_N2-PC		-1619.84 kcal/mol	NIMAG = 0
C	-0.311517	-6.183959	-0.260796
C	-0.472879	-4.658572	-0.311129
N	0.526020	-3.934041	0.454700
H	-0.332792	-6.538972	0.776069
H	0.633693	-6.513135	-0.708244
H	-1.124119	-6.675063	-0.812928
H	-1.449944	-4.390481	0.106655
H	-0.498635	-4.344110	-1.377138
Cl	-0.771728	-0.881551	0.932856
C	1.794778	-3.749318	-0.224131
H	2.362942	-4.688817	-0.286945
H	2.398621	-3.035071	0.344921
H	1.698738	-3.350676	-1.256521
H	0.141885	-3.002317	0.684070

H_3CHN^- E2-PC	-1608.88 kcal/mol	NIMAG = 0	
C	-1.726058	-5.019913	0.521541
C	-2.487147	-5.161035	1.608588
N	-2.231204	-10.079979	-4.759882
H	-1.761727	-5.714317	-0.320267
H	-2.075472	-9.438746	-3.965043
H	-1.025741	-4.186470	0.440710
H	-3.188580	-5.989366	1.700604
H	-2.442153	-4.463825	2.445664
Cl	-1.714148	-7.511167	-2.448652
C	-3.511539	-9.744170	-5.383983
H	-3.684893	-10.380619	-6.264922
H	-3.620491	-8.690506	-5.699617
H	-4.321395	-9.948853	-4.672274
H	-1.484557	-9.840177	-5.410167

$H_3CHNC_2H_5$	-1526.75 kcal/mol	NIMAG = 0	
N	0.672212	1.740363	-0.438252
H	1.511689	1.618176	0.122223
H	-0.108003	-0.260963	-0.172308
C	0.249976	0.442085	-0.948727
H	-0.564308	0.577258	-1.670443
H	1.083366	-0.030750	-1.478283
H	1.019950	3.580674	1.608633
C	-0.340883	2.413507	0.370780
H	-0.709492	1.779514	1.202306
H	-1.208386	2.603229	-0.275823
C	0.169898	3.736165	0.933142
H	0.494204	4.404637	0.129466
H	-0.616903	4.238066	1.505862

H_3CHNH	-798.12 kcal/mol	NIMAG = 0	
N	0.821835	1.580406	-0.102484
H	1.353245	1.089572	0.612274
H	-0.413590	-0.148120	-0.603751
C	0.247393	0.616259	-1.046255
H	-0.327641	1.156337	-1.806260
H	1.058663	0.096086	-1.567029
H	0.077274	2.065608	0.391885

H_3CO^-	-578.86 kcal/mol	NIMAG = 0	
C	-0.393326	-1.822089	-0.000001
H	0.418639	-1.000935	0.000000
O	-1.649699	-1.416181	-0.000001
H	-0.056789	-2.472538	0.892881
H	-0.056791	-2.472537	-0.892880

H_3CO^- RC	-1459.44 kcal/mol	NIMAG = 0	
C	-4.002171	0.128320	0.378429
C	-2.644193	0.030146	-0.230328
Cl	-1.448199	1.417592	0.235087
H	-3.957964	0.104622	1.473205

H	-4.592760	-0.779483	0.013892
H	-4.523787	1.039424	0.063834
H	-2.664842	0.083472	-1.319916
H	-2.108813	-0.868935	0.078284
C	-6.076620	-3.005597	0.158022
H	-5.817548	-4.110204	0.111283
O	-5.373190	-2.216516	-0.671401
H	-5.972241	-2.768390	1.265885
H	-7.203233	-3.001100	0.003428

H_3CO^- S_N2-TS	-1455.47 kcal/mol	NIMAG = 1	$-303.9 cm^{-1}$
C	-1.027401	-5.644284	0.589183
C	-0.740844	-4.438364	-0.254326
O	1.530875	-4.779522	-0.037585
H	-1.596109	-5.372549	1.484094
H	-0.072205	-6.086301	0.889203
H	-1.608708	-6.385478	0.031669
H	-0.430996	-3.524579	0.223551
H	-0.442794	-4.573408	-1.280111
Cl	-2.740272	-3.712655	-0.744798
C	2.262806	-3.843997	-0.689702
H	2.089408	-3.786895	-1.803987
H	3.369570	-4.008708	-0.595233
H	2.113481	-2.782051	-0.335990

H_3CO^- E2-TS	-1458.73 kcal/mol	NIMAG = 1	$-337.6 cm^{-1}$
C	-4.016635	0.340286	0.056039
C	-2.822318	-0.346609	0.519366
Cl	-1.160330	0.825399	0.830173
H	-4.393640	1.076839	0.774625
H	-4.954025	-0.460438	-0.098902
H	-3.886860	0.811677	-0.924736
H	-2.374666	-1.042433	-0.187948
H	-2.882853	-0.775008	1.518254
C	-5.823292	-2.553353	-0.046397
H	-5.931817	-3.259954	-0.914656
O	-6.139754	-1.260756	-0.328751
H	-4.760393	-2.699482	0.294491
H	-6.439371	-3.014914	0.773418

H_3CO^- S_N2-PC	-1493.64 kcal/mol	NIMAG = 0	
C	-0.620346	-5.422319	0.179309
C	-0.071261	-4.004486	0.198811
O	1.344668	-3.887112	0.467436
H	-0.423258	-5.930702	1.131054
H	-0.195140	-6.023128	-0.632623
H	-1.704757	-5.358914	0.016076
H	-0.552038	-3.435611	1.000712
H	-0.309655	-3.495531	-0.746862

Cl	-4.130160	-4.260446	-0.744048
C	2.165278	-4.220677	-0.626579
H	2.109425	-5.286828	-0.901049
H	3.198489	-3.996095	-0.336094
H	1.917338	-3.626817	-1.524367

H_3CO^- E2-PC	-1494.60 kcal/mol	NIMAG = 0	
C	-4.091943	3.692278	0.263092
C	-3.544376	2.819036	1.110025
Cl	-4.526173	-0.953972	1.515784
H	-3.767460	4.731577	0.208505
H	-2.743123	3.126566	1.782729
H	-4.892633	3.395368	-0.412680
H	-3.863022	1.778111	1.170979
C	-5.733380	-4.124943	-0.023171
H	-4.869810	-3.948150	-0.689750
O	-5.422496	-3.940936	1.334117
H	-6.058266	-5.166756	-0.163227
H	-6.550872	-3.467012	-0.369952
H	-5.129106	-2.993732	1.442320

$H_3COC_2H_5$	-1405.58 kcal/mol	NIMAG = 0	
H	0.039807	-0.580103	2.794508
H	-0.708839	-1.523542	1.490216
O	1.994774	0.436105	1.286357
H	0.940457	-1.902811	2.026594
H	0.945885	-0.637105	-0.149406
H	0.044085	0.686946	0.619198
C	0.231778	-1.088884	1.844738
C	0.774744	-0.118682	0.810520
C	2.573115	1.356039	0.381189
H	2.817193	0.883831	-0.584946
H	3.497212	1.719905	0.839065
H	1.909569	2.215418	0.188511

H_3COH	-677.63 kcal/mol	NIMAG = 0	
C	-0.295043	-1.832265	0.009822
H	0.419538	-0.995920	-0.019236
O	-1.648874	-1.391087	-0.057020
H	-0.096047	-2.437627	0.906982
H	-0.125099	-2.458646	-0.870493
H	-1.817017	-0.836041	0.714016

H_3CS^-	-538.95 kcal/mol	NIMAG = 0	
C	-0.237898	-1.872266	-0.000004
H	0.474523	-1.029151	-0.000022
S	-1.981656	-1.309018	0.000069
H	0.003567	-2.486934	0.884454
H	0.003498	-2.486911	-0.884497

H_3CS^- RC	-1415.09 kcal/mol	NIMAG = 0	

C	-2.603412	-5.734341	0.365541
C	-1.371483	-6.291696	1.035128
S	-0.595577	-5.946415	-2.925454
H	-3.450296	-6.424366	0.443258
H	-2.346898	-5.594859	-0.696375
H	-2.893571	-4.769943	0.796249
H	-1.078454	-7.255616	0.619121
H	-0.523170	-5.610520	0.965973
Cl	-1.603505	-6.593373	2.838334
C	0.257157	-4.431086	-3.501768
H	1.350375	-4.563831	-3.541894
H	0.065069	-3.567058	-2.845026
H	-0.065468	-4.138935	-4.514245

H_3CS^- S_N2-TS	-1408.23 kcal/mol	NIMAG = 1	$-308.2 cm^{-1}$
C	-1.248206	-5.843017	0.091811
C	-0.896512	-4.385393	-0.012282
S	1.733641	-4.703010	-0.524982
H	-1.660030	-6.067545	1.080241
H	-0.367419	-6.471506	-0.066691
H	-2.002106	-6.108976	-0.655127
H	-0.460351	-3.874219	0.827599
H	-0.816962	-3.916923	-0.977226
Cl	-2.931361	-3.445326	0.367619
C	2.208431	-2.946707	-0.648855
H	2.013568	-2.400283	0.285141
H	1.668688	-2.429837	-1.455323
H	3.281986	-2.857103	-0.863093

H_3CS^- E2-TS	-1404.94 kcal/mol	NIMAG = 1	$-602.9 cm^{-1}$
C	-2.395401	-5.562192	-0.165941
C	-1.305509	-5.224922	0.641842
S	-1.622834	-5.944663	-3.095156
H	-2.828683	-6.553521	-0.021237
H	-1.979556	-5.725543	-1.499021
H	-3.142851	-4.785106	-0.335716
H	-0.599504	-5.990034	0.943814
H	-0.915221	-4.213753	0.628233
Cl	-1.745138	-4.877235	3.034488
C	0.137703	-5.572251	-2.824675
H	0.772807	-6.423147	-3.097711
H	0.309131	-5.349556	-1.762219
H	0.455821	-4.700122	-3.407860

H_3CS^- S_N2-PC	-1440.49kcal/mol	NIMAG = 0	
C	-0.674426	-5.691656	0.744313
C	-0.159038	-4.277489	0.486617
S	1.650455	-4.121429	0.276717
H	-0.202046	-6.144982	1.623717
H	-0.497295	-6.337808	-0.120104
H	-1.760283	-5.663947	0.905754
H	-0.383648	-3.625693	1.339930
H	-0.635932	-3.854094	-0.405912
Cl	-1.526823	-3.684958	-3.049416

C	1.861187	-4.636151	-1.457054
H	2.833674	-4.250042	-1.778300
H	1.064433	-4.209190	-2.076499
H	1.862078	-5.726126	-1.557108

H_3CS^- E2-PC	-1428.25 kcal/mol	NIMAG = 0	
C	-2.341573	-4.907482	0.392211
C	-1.509344	-5.024762	1.428122
S	-4.061700	-9.911205	-4.811836
H	-2.571027	-5.736871	-0.276503
H	-3.877778	-9.032561	-3.720976
H	-2.830064	-3.956886	0.176794
H	-1.016680	-5.969736	1.652032
H	-1.286435	-4.189241	2.091721
Cl	-3.383624	-7.728650	-2.183059
C	-2.322248	-9.858373	-5.351213
H	-2.218658	-10.498749	-6.233050
H	-1.658569	-10.225355	-4.562717
H	-2.026851	-8.838129	-5.613258

$H_3CSC_2H_5$	-1347.82 kcal/mol	NIMAG = 0	
C	-0.631255	-5.700752	0.722446
C	-0.187229	-4.257777	0.491648
S	1.598004	-3.990353	0.231412
H	-0.104431	-6.143840	1.572611
H	-0.450716	-6.329845	-0.154097
H	-1.707798	-5.731745	0.932497
H	-0.407072	-3.648164	1.375361
H	-0.730794	-3.806426	-0.347092
H	1.684738	-5.728669	-1.490509
C	1.845595	-4.648557	-1.440195
H	2.888619	-4.441180	-1.694000
H	1.201957	-4.140307	-2.165418

H_3CSH	-614.83 kcal/mol	NIMAG = 0	
C	-0.256971	-1.899117	-0.039617
H	0.332463	-0.985078	-0.140150
S	-2.041915	-1.535798	0.036858
H	-0.007372	-2.375248	0.911742
H	-0.025207	-2.592576	-0.851035
H	-2.143018	-0.969063	-1.181076
